# Constructing neural networks with pre-specified dynamics

**DOI:** 10.1038/s41598-024-69747-z

**Published:** 2024-08-14

**Authors:** Camilo J. Mininni, B. Silvano Zanutto

**Affiliations:** 1 Instituto de Biología y Medicina Experimental, Consejo Nacional de Investigaciones Científicas y Técnicas, Buenos Aires, Argentina; 2 Instituto de Ingeniería Biomédica, Universidad de Buenos Aires, Buenos Aires, Argentina

**Keywords:** Neural networks, Brain dynamics, Model fitting, Dynamical systems, Network models, Neural circuits

## Abstract

A main goal in neuroscience is to understand the computations carried out by neural populations that give animals their cognitive skills. Neural network models allow to formulate explicit hypotheses regarding the algorithms instantiated in the dynamics of a neural population, its firing statistics, and the underlying connectivity. Neural networks can be defined by a small set of parameters, carefully chosen to procure specific capabilities, or by a large set of free parameters, fitted with optimization algorithms that minimize a given loss function. In this work we alternatively propose a method to make a detailed adjustment of the network dynamics and firing statistic to better answer questions that link dynamics, structure, and function. Our algorithm—termed generalised Firing-to-Parameter (gFTP)—provides a way to construct binary recurrent neural networks whose dynamics strictly follows a user pre-specified transition graph that details the transitions between population firing states triggered by stimulus presentations. Our main contribution is a procedure that detects when a transition graph is not realisable in terms of a neural network, and makes the necessary modifications in order to obtain a new transition graph that is realisable and preserves all the information encoded in the transitions of the original graph. With a realisable transition graph, gFTP assigns values to the network firing states associated with each node in the graph, and finds the synaptic weight matrices by solving a set of linear separation problems. We test gFTP performance by constructing networks with random dynamics, continuous attractor-like dynamics that encode position in 2-dimensional space, and discrete attractor dynamics. We then show how gFTP can be employed as a tool to explore the link between structure, function, and the algorithms instantiated in the network dynamics.

## Introduction

Neural network models play a crucial role in neuroscience, as they enable the formulation of explicit hypotheses that establish connections between behaviour and neurophysiology. Networks can be constructed from the bottom up, taking experimental evidence to define the neuron input-output mapping and their connectivity^1,2^. Then, the emergent properties of the system can be studied, expecting that they recapitulate experimental observations not used as model hypotheses. On the other hand, a normative approach is also possible, in which we start from the function the network is proposed to have, together with a few assumptions about network connectivity and neuron activation dynamics, and then we fit the free parameters of the model to obtain the best task performance, as measured by a loss function. This approach has increasingly gained momentum due to advances in deep learning that have provided researchers with new tools and hardware to fit increasingly complex models to increasingly complex tasks^3^. The models obtained have reproduced experimental observations regarding the nature of neural coding, distributed representation and population dynamics^4–8^. Modelling approaches of this kind stem from a paradigm that proposes to understand neural computation as algorithms instantiated in the low dimensional dynamics of large neural populations^9,10^. With neural recording technology allowing more simultaneous measurements^11^, fitting neural networks with many neurons, capable of complex dynamics is going to be more and more necessary. Nonetheless, network optimization is a complex endeavour. The way fitted networks solve the task and represent information depends on the choice of all hyperparameters, hindering our ability to understand how each hypothesis influences networks behaviour. A fitting algorithm may converge preferentially to a specific family of solutions, giving no information about how many other networks there are that can solve the task in qualitatively different ways. The existence of multiple synaptic weight configurations that lead to the same network output makes it difficult to relate network function to its connectivity^12^. Furthermore, it is possible that a low error network exists, yet the optimization algorithm fails to find it. Training is usually easier if networks have many neurons (with many parameters), hence there is a bias towards choosing networks with more neurons than the minimum required, making the number of weight configurations even larger. However, some of these shortcomings can be handled by leveraging the particular goals of modelling in neuroscience. First, we expect our network to solve tasks like the ones solved by animals in behavioural experiments. These tasks are usually easy to solve, in the sense that we already know how to solve them. We can even enumerate more than one algorithm that solves the same task. Second, we usually have some hypotheses about how the modelled system represents and processes information. For instance, we may have evidence that neural populations in the prefrontal cortex handle working memory demands by instantiating discrete attractors that encode relevant stimuli^13,14^, or that networks in the entorhinal cortex encode position in a 2D continuous attractor^15–17^. Therefore, it would be of interest to have a network that solves a task through a given a priori dynamics, and then study how the underlying connectivity relates to function. This approach could be achieved, at least in principle, by algorithms that maximise some target emergent properties that can be quantified and included in the loss function, or with algorithms that find an approximation to a target dynamics that is low dimensional, like in the 1D or 2D attractor examples mentioned above. However, as the constraints on the target dynamics increase, the optimization problem becomes harder. In a previous work we presented an algorithm to construct recurrent neural networks that follow a target dynamics^18^. Networks were composed of binary neurons^19–21^, and their dynamics were specified in the form of a graph—termed the *transition graph *—in which each node represents a network activation state, and each arc a transition between states triggered by a given stimulus the network receives as input. This approach is closely related to previous work that connected neural networks with finite state machines^22–28^. For instance, Minsky showed that any finite state machine can be represented in a neural network by assigning one neuron to each of the machine’s (input, state) pairs, and connecting neurons according to the machine’s transition function^22^. Later, Alquézar and Sanfeliu^29^ gave a formal proof of Minsky’s construction, and generalised it to neural networks with orthogonal inputs and network states. Although this method allows great control of network dynamics, the requirements of one neuron per (input, state) pair and the use of orthogonal representations reduce its appeal for neuroscience modelling applications. In the present work we address these limitations by introducing a new algorithm, termed *generalised Firing-to-Parameter* (gFTP), which constructs neural networks that unfold a specific user-define dynamics. The algorithm takes a transition graph as input, decides if there is a neural network capable of following the transition graph exactly, and if not, expands the original graph to construct a new graph that fulfils two conditions: it is realisable in terms of a neural network, and it retains all of the information present in the original graph. Then, we define the network firing states associated with each node in the graph, in such a way that they comply with a set of linear constraints that directly stem from the graph structure. Finally, weight matrices are found with the accelerated perceptron algorithm^30^.

In the following, we start by giving a detailed step-by-step description of the rationale behind gFTP, through a set of simple examples. Next, we evaluate its performance on random transition graphs, graphs instantiating a discretised version of a 2-dimensional continuous attractor that encodes position, and graphs instantiating a discrete attractor dynamics. Then, we show how gFTP can be employed in combination with an optimization algorithm as an exploratory tool to disentangle the multiple dependencies between structure and function. Finally, we analyse the underlying differences in dynamics and connectivity subserving two different ways of solving the same behavioural task.

## The generalised firing to parameter algorithm

We will introduce an algorithm that constructs a neural network that follows a given user-defined dynamics. If this is not possible, the algorithm finds a new equivalent dynamics that preserves the encoded information. We will first describe the neural network model, how the dynamics is specified, and the conditions necessary for the dynamics to be realisable by a neural network (see Table 1 in “Methods” for a quick reference to symbols and their definitions).

### Network model and consistency conditions

**Figure 1:**
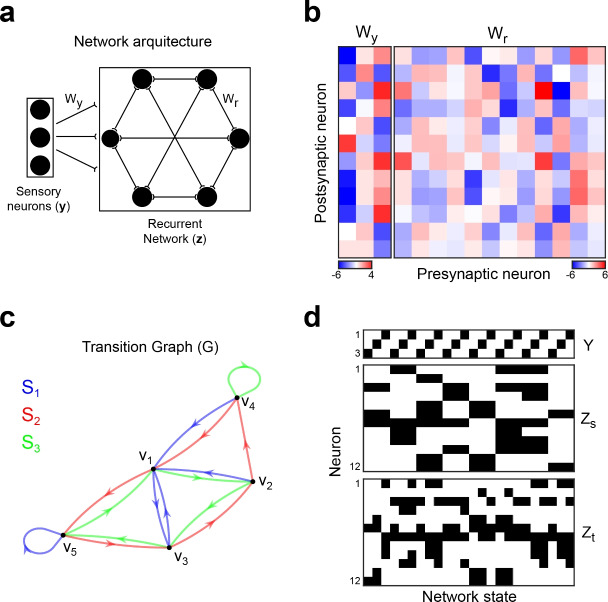
Recurrent neural networks that follow a pre-specified transition graph. (**a**) Recurrent networks of binary neurons receive connections from neurons that encode stimuli in a one-hot fashion. (**b**) Example of synaptic weight matrices $\textbf{W}_{y}$ and $\textbf{W}_{r}$. The network is composed of 3 sensory neurons and 12 recurrently connected neurons. (**c**) Example of transition graph that defines the desired network dynamics. Each node represents a different network activation state. Directed arcs depict transitions from source to target states that are triggered by different stimuli, encoded by the sensory neurons and shown with arrows of different colours. (**d**) Example matrices $\textbf{Y},$ $\textbf{Z}_{s}$, and $\textbf{Z}_{t}$.

We will consider recurrent neural networks composed of binary neurons, whose activations can take values 0 or 1 (Fig. 1a). Neurons receive inputs from sensory neurons and from all other neurons in the network.

The network state at iteration *k* is dictated by the map:

1 $$\begin{aligned} \mathbf { \textbf{u} }(k)&=\textbf{W}_{y}\textbf{y}(k)+\textbf{W}_{r}\textbf{z}(k-1) \end{aligned}$$

2 $$\begin{aligned} \textbf{z}(k)&=\mathcal{\text {H}} (\textbf{u}(k)) \end{aligned}$$

where $\textbf{u}$ is the $N_{neu}$-dimensional vector of network preactivations, $\textbf{y}$ is the $N_{s}$-dimensional vector encoding the presence of $N_{s}$ different stimuli in a one-hot fashion, $\textbf{z}$ is the $N_{neu}$-dimensional vector of network activations, and $\mathcal {\text {H}}$ the Heaviside function. The ith row vector in matrix $\textbf{W}_{y}$ collects the synaptic weights of the connections from stimulus to the ith neuron in the recurrent network, while the ith row vector in matrix $\textbf{W}_{r}$ collects the synaptic weights of the connections the ith neuron receives from all neurons in the network (Fig. 1b).

We want to find matrices $\mathbf {W_{ y }}$, $\mathbf {W_{ r }}$ and a set of network activation vectors $Z=\{\textbf{z}_{v}\}$ such that stimuli trigger transitions between these network activations in accord with a desired, user-specified target trajectory in state space. We will specify a target trajectory as a multi digraph, in which each node *v* in the graph represents a network activation state $\textbf{z}_{v}$, and the arcs represent transitions triggered by stimuli. Formally, we define a labelled multi digraph $G=(V,K,S,f_{s},f_{t},l_{G})$, where *V* is the set of $N_{v}$ nodes $v_{1,}v_{2,}\ldots v_{N_{v}}$, $K\subset \mathbb {N}$ is the set of transitions specified (the arcs in the graph), $S\subset \mathbb {N}$ is the set of stimuli (the arc’s labels) that act as the network’s inputs, $f_{s}$ is the function that takes an arc and gives its source node, $f_{t}$ is the function that takes an arc and gives a target node, and $l_{G}$ the function that takes an arc and gives its label (the stimulus that triggers that transition) (Fig. 1c). Equivalently, a matrix $\textbf{G}$ can be constructed, such that row *i* in $\textbf{G}$ is of the form $(l_{G}(k),f_{s}(k),f_{t}(k))$, specifying the ith transition. Each node $v\in V$ will be encoded by its unique and specific vector **z**.

We define matrices $\textbf{Y}$, $\textbf{Z}_{s}$ and $\textbf{Z}_{t}$ as the matrices that collect in each of their column the activations of the sensory neurons ($\textbf{Y}$), and the activation of the recurrent network in a source state ($\textbf{Z}_{s})$ and in a target state ($\textbf{Z}_{t}$), for each transition/column *k* (i.e. $\textbf{Y}(:,k)=\textbf{y}_{\textbf{G}(k,1)}$, $\textbf{Z}_{s}(:,k)=\textbf{z}_{\textbf{G}(k,2)}$ and $\textbf{Z}_{t}(:,k)=\textbf{z}_{\textbf{G}(k,3)},$ with $\textbf{y}_{i}$ being the input when the network is receiving stimulus *i*, and $\textbf{z}_{i}$ being the activation state when the network state is $v_{i}$ (Fig. 1d)). A network that instantiates graph *G* must satisfy:

3 $$\begin{aligned} \textbf{W}\,\textbf{C}=\textbf{U},\,\,\,\,s.t.\, {\mathcal{\text{H}}}(\textbf{U})=\textbf{Z}_{t}, \end{aligned}$$

with

4 $$\begin{aligned} \textbf{C}&=\begin{pmatrix}\textbf{Y}\\ \textbf{Z}_{s} \end{pmatrix}\end{aligned}$$

5 $$\begin{aligned} \textbf{W}&=\begin{pmatrix}\textbf{W}_{y},\textbf{W}_{r}\end{pmatrix} \end{aligned}$$

This is a problem of linear separability. Matrix $\textbf{W}$ exists iff two conditions are met: 1. $rank(\textbf{C})=rank((\textbf{C}^{T},\textbf{U}^{T}))$ (i.e. the augmented matrix $(\textbf{C}^{T},\textbf{U}^{T})$ and matrix $\textbf{C}$ present the same linear combinations), and 2. the signs of values in **U** are consistent with $\textbf{Z}_{t}$. If $\textbf{Z}_{s}$ and $\textbf{Z}_{t}$ are chosen properly, then a $\textbf{W}$ that is a solution for Eq. (3) exists and can be found with recently proposed accelerated versions of the perceptron algorithm^30^. Finding suitable $\textbf{Z}_{s}$ and $\textbf{Z}_{t}$ matrices is a hard combinatorial problem. However, we can leverage some regularities to reduce its complexity. For a start, we must decide if such $\textbf{Z}_{s}$, $\textbf{Z}_{t}$ exist in the first place, since it could be the case that, for some transition graphs, the interdependencies between source and target nodes preclude its instantiation in the shape of any neural network. In this sense, we must find out if the graph is realisable, and if not, we must find a way to turn the graph into a realisable graph, without losing the critical aspects of the target dynamics.

There is a set of linear combinations that directly stems from the transition graph, and can be detected and corrected if needed. Let’s consider two transitions that start from the same source node *v*, but are triggered by different stimuli:

6 $$\begin{aligned}{} & {} \begin{pmatrix}\textbf{W}_{y},\textbf{W}_{r}\end{pmatrix}\begin{pmatrix}\textbf{y}_{s_{i}}\\ \textbf{z}_{v} \end{pmatrix}=\textbf{u}_{s_{i},v} \end{aligned}$$

7 $$\begin{aligned}{} & {} \begin{pmatrix}\textbf{W}_{y},\textbf{W}_{r}\end{pmatrix}\begin{pmatrix}\textbf{y}_{s_{j}}\\ \textbf{z}_{v} \end{pmatrix}=\textbf{u}_{s_{j},v} \end{aligned}$$

where $\textbf{u}_{s_{i},v}$, $\textbf{u}_{s_{j},v}$ are the preactivations after the network receives stimulus $s_{i}$ or $s_{j}$, starting from the same node *v*. Subtracting both sides of the equations we get:

8 $$\begin{aligned} \begin{pmatrix}\textbf{W}_{y},\textbf{W}_{r}\end{pmatrix}\begin{pmatrix}\textbf{y}_{s_{i}}-\textbf{y}_{s_{j}}\\ \textbf{z}_{v}-\textbf{z}_{v} \end{pmatrix}&=\begin{pmatrix}\textbf{u}_{s_{i},v}-\textbf{u}_{s_{j},v}\end{pmatrix} \end{aligned}$$

9 $$\begin{aligned} \textbf{W}_{y}\left( \textbf{y}_{s_{i}}-\textbf{y}_{s_{j}}\right)&=\begin{pmatrix}\textbf{u}_{s_{i},v}-\textbf{u}_{s_{j},v}\end{pmatrix} \end{aligned}$$

Since both transitions start from the same node, the inputs that a neuron receives that come from the other neurons in the recurrent network cancel, and the difference in $\textbf{u}$ only depends on the input from the stimuli. Vector $\mathbf {\varvec{\delta }}_{i,j}:=\textbf{W}_{y}(\textbf{y}_{s_{i}}-\textbf{y}_{s_{j}})$ is a constant that does not depend on the source node *v*. Hence, vector $\varvec{\delta }\textbf{u}_{i,j,v}:=\textbf{u}_{s_{i},v}-\textbf{u}_{s_{j},v}$ does not depend on *v* either, since it must be equal to $\mathbf {\varvec{\delta }}_{i,j}$, regardless of *v*. For a given neuron *n*:

10 $$\begin{aligned} (\textbf{z}_{s_{i},v}(n),\textbf{z}_{s_{j},v}(n))\in \{(1,0),(0,0),(1,1)\}\Leftrightarrow \textbf{z}_{s_{i},v}(n)\ge \textbf{z}_{s_{j},v}(n)&\Leftrightarrow \textbf{u}_{s_{i},v}(n)-\textbf{u}_{s_{j},v}(n)\ge 0 \end{aligned}$$

11 $$\begin{aligned} (\textbf{z}_{s_{i},v}(n),\textbf{z}_{s_{j},v}(n))\in \{(0,1),(0,0),(1,1)\}\Leftrightarrow \textbf{z}_{s_{i},v}(n)\le \textbf{z}_{s_{j},v}(n)&\Leftrightarrow \textbf{u}_{s_{i},v}(n)-\textbf{u}_{s_{j},v}(n)<0 \end{aligned}$$

**Figure 2:**
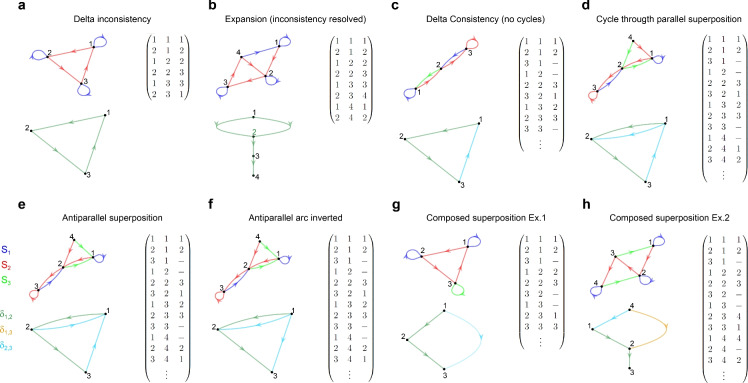
Example graphs *G* (upper left of each panel), matrix $\mathrm {\textbf{G}}$ (upper right of each panel, with columns showing stimulus, source node and target node), and their associated graph *D* (lower left of each panel). (**a**) Graph *D* forming a cycle of 3 nodes. If a neuron is active in one node and not active in the other node, we arrive at an inconsistency. (**b**) Inconsistency in (**a**) is resolved by adding node $v_{4}$ with same targets as $v_{1}$ (node $v_{1}$ is expanded). The associated graph *D* has no cycles. (**c**) A graph with 3 nodes and 3 stimuli (dashes stand for unassigned target nodes). Graph *D* has no cycles because it is not possible to flow from a green arc to a cyan arc. (**d**) Green and cyan arcs in graph *D* are superimposed, putting them in the same traversable set. Hence, there is a cycle. (**e**) Green and cyan arcs in graph *D* are superimposed but they have opposite directions. These deltas are linked, but arcs labelled with one of these deltas must be inverted. (**f**) Same graph *D* as in (**e**), but cyan arcs have been inverted. All arcs belong to the same traversable set, but no cycles are present. Graph *G* is consistent. (**g**) Arcs green and cyan are not directly superimposed but they are linked: defining $\delta _{1,2}$ or $\delta _{2,3}$ also defines $\delta _{1,3}$. **(h)** Composed superposition appears any time two nodes are reachable by more than one path (regardless of arcs direction).

Relations (10, 11) show that activation states of target nodes reached from the same source node determine one delta value for each pair of stimuli and for each neuron. In other words, each neuron will have a delta value for each pair of stimuli, and this value is a constant that must by fulfilled by the activation states in target nodes that come from a common source node. For instance, a pair $(\textbf{z}_{s_{i},v_{1}}(n),\textbf{z}_{s_{j},v_{1}}(n))$ adopting values (1, 0) implies that $\delta _{i,j}\ge 0$ for neuron *n*, meaning that assigning (0, 1) to the pair $(\textbf{z}_{s_{i},v_{2}}(n),\textbf{z}_{s_{j},v_{2}}(n))$ results in an inconsistency, regardless of the identity of nodes $v_{1},v_{2}$. Not taking this fact into consideration will lead to $\textbf{Z}_{s}$ and $\textbf{Z}_{t}$ that are not realisable in any neural network. Moreover, it could be the case that the graph itself does not allow *any* $\textbf{Z}_{s}$, $\textbf{Z}_{t}$ to be realisable. Consider for example the upper graph in Fig. 2a (and its associated matrix $\textbf{G}$). If a neuron differentiates $v_{1}$ from $v_{2}$, by firing when the network is in state $v_{1}$ ($z_{v_{1}}=1$) and not firing in state $v_{2}$ ($z_{v_{2}}=0$), then $\delta _{1,2}>0$. This in turn means that the neuron must not fire in state $v_{3}$ ($z_{v_{3}}=0$), because if it fired then $\delta _{1,2}<0$, leading to a contradiction to the value of delta defined by transitions 1 and 2. We say that the value of zero assigned to $v_{2}$ “propagates” to $v_{3}$. This value propagates from $v_{3}$ to $v_{1}$ too ($z_{v_{3}}=z_{v_{1}}=0$), where we started. But this is in contradiction with the original value for $z_{v_{1}}$. The inconsistency is circumvented only when $v_{1}$ and $v_{2}$ are not differentiated by any neuron, but in this case there would be only two network states instead of three. Therefore, the graph is unrealisable as it is. However, the graph can be modified to make it realisable. Let’s consider the modified graph in Fig. 2b. We have replaced $v_{1}$ in transition 6 with a new node ($v_{4}$), and added transitions for this new node as source, equal to the transitions of the node it replaced (transitions with $v_{1}$ as a source node). The new node encodes the same information as the replaced node, i.e. the occurrence of stimulus $s_{2}$ starting from $v_{3}$. It also leads to the same transitions too (going to $v_{1}$ through $s_{1}$, and going to $v_{2}$ through $s_{2}$). Therefore, the new graph (Fig. 2b) is realisable, and all the information encoded in the network states is preserved. It is important to recall that the contradiction in delta values that takes place in the graph of Fig. 2a occurs because delta propagates, and because node $v_{1}$ appears twice as target, once triggered by $s_{1}$ and another by $s_{2}$. On the other hand, since the new node $v_{4}$ only appears once as target, its addition cannot lead to a new contradiction. The new node is a twin of the node it replaces, in the sense that it leads to the same targets through the same stimuli. We will say that a transition graph is *delta consistent *if there are $\textbf{Z}_{s}$, $\textbf{Z}_{t}$ and $\textbf{U}$ such that relations (10, 11) are satisfied. If this is not the case (because differentiating some nodes leads to a conflict with relations (10, 11)) then the graph is termed *delta inconsistent*. We will name “expansion” to the action of replacing a target node with a new node at a set of conflicting transitions, as we did with the graph of Fig. 2a. Each expansion removes one inconsistency.

### Detecting inconsistencies by searching for cycles in auxiliary graph *D*

In the graph of Fig. 2a we see that the inconsistency appears because we differentiate $v_{1}$ from $v_{2}$, defining $\delta _{i,j}$ in the process. Assigned values “propagate” to other nodes, reaching back to one of the nodes we already defined. Therefore, the inconsistency occurred because we tried to differentiate two nodes that are part of a certain kind of *cycle*. To make this intuition more precise we define a labelled directed multi digraph $D=(V,A,C,g_{s},g_{t},l_{D}),$where *V* is the set of nodes, $A\subset \mathbb {N}$ is the set of arcs, $C=\{\delta _{i,j}:(i,j)\in S\times S\},$ the set of labels for the arcs, $g_{s}$ the function that takes an arc and gives its source node, $g_{t}$ the function that takes an arc and gives its target node, and $l_{D}$ the function that takes an arc and gives its label (lower graph of each panel in Fig. 2). An arc *a* from source node $v_{i}$ to target node $v_{j}$ exists in *D* if $v_{i}\not =v_{j}$, and there exist arcs $a_{1},a_{2}$ and source node $v_{s}$ in *G* such that $f_{t}(a_{1})=v_{i},\,f_{t}(a_{2})=v_{j}$ and $f_{s}(a_{1})=f_{s}(a_{2})=v_{s}$. This arc will have label $l_{D}(a)=\delta _{m,n}$, being *m* and *n* the stimuli through which $v_{i}$ and $v_{j}$ are reached, respectively. In other words, two nodes are connected by an arc in *D* if in graph *G* these nodes are target nodes of the same source node. The label of the arc is $\delta _{m,n}$, the delta associated with the stimuli that lead to each of the connected nodes. The direction of the arc is given by the sign of $\delta _{m,n}$. Self loops ($g_{s}(a)=g_{t}(a)$) are not included in *D* because they represent cases in which $\delta =0$, which does not constrain assignments to *z* in any way.

Now we can understand inconsistencies in terms of graph *D*: if it is possible to go from one node of graph *D* to another node, travelling arcs of the *same label*/*delta*, and reach the node from where we started, then graph *G* has at least one inconsistency. This is because nodes in *D* are connected if they are linked by deltas, and the direction of the arc is defined by the delta sign, which means that assigning different *z* values to any pair of nodes within this kind of cycle will necessarily lead to a contradiction of deltas, as relations (10, 11) will not hold. Therefore, we can decide if an inconsistency exists by exploring *D* in search of cycles formed by arcs of the same label (simply referred to as cycles from now on). If any such cycle exists, then there is at least a pair of nodes that cannot be differentiated without getting a delta inconsistency. To assure delta consistency, we must break all these cycles by breaking any of their arcs. Since an arc exists when two nodes are targets of the same source node, the arc can be broken by expanding one of the nodes of the arc, at all transitions in *G* that allow the arc to exist. The expansion breaks the cycle. We have to repeat the process of cycle search and expansion until no cycles are left. The final graph will be delta consistent, and will hold all the information encoded by the original graph.

### Detecting equivalent labels

So far we have considered the example of Fig. 2a in which there are only 2 stimuli and hence one delta. Let’s now consider a graph with 3 stimuli and 2 deltas. In Fig. 2c we can see a subset of a transition graph (some target nodes are left unassigned) which shows no cycles, hence nodes $v_{1}$, $v_{2}$ and $v_{3}$ can be differentiated. However, in the graph of Fig. 2d, node $v_{1}$ and $v_{2}$ are connected by two arcs with different labels. Differentiating these two nodes will define two deltas, and they will have the same sign. We call *Arc label superposition* when two or more deltas are linked in this way, since defining one delta necessarily defines the other. Arcs with superimposed labels are equivalent in the sense that they can be traversed as if they had the same label, and should be treated as if they were of equal label while searching for cycles.

Arc superposition can be parallel, like in the example above (superimposed arcs have the same direction), but can also be antiparallel (arcs in opposite directions). In this case, differentiating $v_{1}$ from $v_{2}$ defines two deltas, but the graph cannot be traversed in the same way as when superposition goes in the same direction. Consider the graph in Fig. 2e, which is equal to the previous graph, except for the last two transitions, where the target nodes were switched. In this case, it is easy to check that no inconsistency occurs. Yet, a cycle would be detected if the algorithm so far described is executed. This issue is solved by noting that an antiparallel superposition implies that deltas have opposite signs, meaning that the graph should be traversed in opposite directions for arcs of superimposed labels. Hence, for each case of antiparallel superposition we flip the direction of all arcs that share the label with one of the antiparallel superimposed arcs (Fig. 2f), with the precaution of only flipping arcs that weren’t already flipped.

Finally, we can see that the parallel and antiparallel superpositions shown so far are particular examples of a broader definition of superposition. Consider the graph in Fig. 2g. In this case, differentiating $v_{1}$ from $v_{2}$ defines $\delta _{1,2}$ but also $\delta _{1,3}$. In the graph of Fig. 2h differentiating $v_{1}$ from $v_{2}$ defines $\delta _{1,2}$ but also one of the other two deltas: $\delta _{4,2}$ (parallel to $\delta _{1,2}$) or $\delta _{4,1}$ (antiparallel to $\delta _{1,2}$). It is clear that superposition can occur between two arcs that do not share their source and target nodes. We say it is a *composed superposition*. With this in mind, we are able to propose a more general way of detecting arc label superposition. Superimposed deltas can be represented as a partition *P* of the set of labels *C* (i.e. $P=\{\mathcal {C}_{i}\}_{i}$, with $\cap _{i,j}(\mathcal {C}_{i},\mathcal {C}_{j})=\emptyset \; \forall i \ne \ j, \cup _{i}\mathcal {C}_{i}=C ).$ Then, for each element of the partition, there is one *traversable set* of arcs $V_{trav}(\mathcal {C}_{i})=\{(v_{s},v_{t}):l_{D}(a)\in \mathcal {C_{ i }},v_{s}=f_{s}(a),v_{t}=f_{t}(a),a\in A\}.$ We initialise the partition as the finest possible, i.e. $P=\{\{c_i\}\}_i$ with $c_i \in C$. Then, to detect a composed superposition we first partition graph *D* into disjoint complete paths. Each complete path is a path within a traversable set such that the first node in the path is never a target node in the traversable set, and the last node is never a source node in the traversable set. We select one complete path within a given traversable set, taking arc direction into consideration (path 1). Then, we search for a second path (path 2) that connects two nodes of the path 1, but with the condition that (1) we do not take arc direction into consideration for this connection, and (2) arc labels in path 2 are from any traversable set except the traversable set of path 1. If such path 2 exists, then the label of the arcs in path 1 is superimposed to exactly one of the labels of the arcs in path 2, (chosen at random from all labels encountered in path 2). Since for path 2 we did not take arc direction into consideration, superimposed arcs in this path could be traversed in two possible directions. If the direction matches the direction of arcs in path 1, then we have parallel superposition. If the direction is opposite to the direction in path 1, then we are in a case of antiparallel superposition, hence the direction of all arcs in graph *D* labeled by the superimposed label found in path 2 must be inverted. Once the superposition is detected, elements of the partition *P* and the associated traversable sets are merged to account for the new superimposed deltas. Note that this way of detecting composed superposition will also detect simple parallel and antiparallel superpositions as the ones in examples of Fig. 2d, e.

In summary, the process of constructing a consistent transition graph from a given target graph starts by initializing partition *P* as stated above and computing its associated traversable sets. Then, we partition each traversable set into disjoint complete paths and detect the composed superpositions. Partition *P* and traversable sets are modified according to the presence of parallel and antiparallel superpositions. Antiparallel arcs are inverted, but arcs that were flipped once are not flipped again. We merge elements of *P* and traversable sets according to the superpositions encountered. Next, we find cycles in graph *D* by choosing a set $\mathcal {C}$ from *P*, and then a source node from $V_{trav}(\mathcal {C})$. We perform depth first search (DFS), traversing the graph through arcs that are members of $V_{trav}(\mathcal {C})$. When no cycles are encountered within one traversable set, a new $\mathcal {C}$ is chosen and its associated $V_{trav}(\mathcal {C})$ explored. When a cycle is encountered, a node from the cycle is expanded, graph *G* and *D* are updated, and the process is restarted from the superposition detection step. If all traversable sets were explored and no cycles were found, the process stops and returns the final consistent graph $G_{cons}$ (see the Supplementary Information for pseudocode of the gFTP algorithm and its subfunctions).

### Construction of $\textbf{Z}_{s}$ and $\textbf{Z}_{t}$

To construct matrices $\textbf{Z}_{s}$, $\textbf{Z}_{t}$ we must find the activation values for each neuron in the network at each node in the consistent graph, in such a way that each node has its own unique associated activation vector that encodes the node (these constitute the column vectors in $\textbf{Z}_{s}$, $\textbf{Z}_{t}$), and the vector of activation states across transitions satisfies relations (10, 11) for each neuron (each of the row vectors in $\textbf{Z}_{s}$, $\textbf{Z}_{t}$). Finding such activation states is a constraint satisfaction problem which we solve with a backtracking algorithm, where there is one binary decision for each node and the constraint to satisfy is delta consistency. Values 1 or 0 are assigned to nodes, and if delta consistency is sustained, we search for nodes that are obliged to have a certain value, either 1 or 0, such that delta consistency is not violated, i.e. assigned values are “propagated” to other values, according to deltas defined so far (for example, if $\delta _{ij}=u_{1}-u_{2}>0$ and $z_{1}=0$, then $z_{2}$ must be 0 in order to keep delta consistency). Value propagation using delta consistency helps in reducing the number of combinations to try. To reduce it further, nodes are explored in decreasing order of their in-degree in graph $G_{cons}$. This follows from the assumption that nodes that appear more times as targets in *G* have more instances to satisfy a given delta. This should make their assignment more difficult and critical to following assignments, and thus they should be assigned first. Details on our backtracking algorithm for constructing $\textbf{Z}_{s}$ and $\textbf{Z}_{t}$ can be found in the “Methods” section.

### Overview of the gFTP algorithm

1. Graph *D* is constructed from graph *G*.
2. Arc superpositions are detected and traversable sets are updated.
3. Graph *D* is traversed through DFS.
4. If a cycle is found, one of its nodes is expanded.
5. Steps 1 to 4 are repeated until no cycles are present.
6. Matrices $\textbf{Z}_{s}$ and $\textbf{Z}_{t}$ are constructed.
7. Matrices $\textbf{W}_{y}$ and $\textbf{W}_{r}$ are obtained with the accelerated perceptron algorithm.
8. If the perceptron learning error is not zero, new matrices $\textbf{Z}_{s}$ and $\textbf{Z}_{t}$ are constructed and concatenated to the previous ones.
9. Steps 7 and 8 are repeated until step 7 achieves zero error.

**Figure 3:**
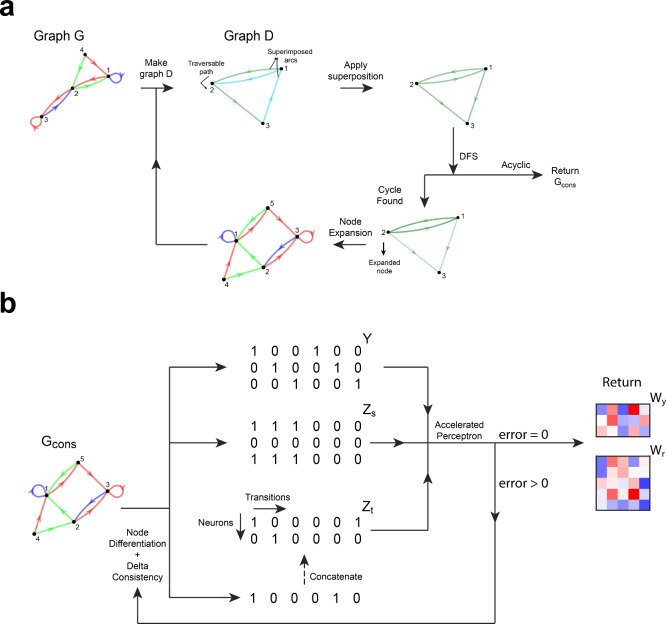
Graphic depiction of gFTP main stages. (**a**) Main steps to attain a consistent transition graph. gFTP takes a user-defined transition graph as input. An auxiliary graph *D* is constructed, which contains information about possible inconsistencies. Arc superpositions are detected (in this case, superposition between a complete path, connecting nodes 1, 2, and 3, and the arc between nodes 1 and 3 (an antiparallel superposition, since we can go from 1 to 3 though green arcs, and from 3 to 1 through a cyan arc). Detected superpositions are applied to traversable sets (all arcs in *D* are now green because they must be treated as if they were of the same label; cyan arcs were inverted because superposition was antiparallel). Depth First Search (DFS) is conducted to search for cycles. The absence of cycles indicates that graph *G* is consistent. Cycle presence requires the expansion of one node in *G* that composes the cycle in *D* after applying superpositions (node 2 was expanded in this case, but expansion of node 1 was also possible). A new cycle of the algorithm is executed starting from the expanded graph. (**b**) Main steps to obtain matrices $\textbf{Z}_{s}$, $\textbf{Z}_{t}$, $\textbf{W}_{y}$, $\textbf{W}_{r}$ from $G_{cons}$. A row vector of ones and zeros is constructed in each iteration (vector’s elements are neuron’s output in each transition). This vector must differentiate at least two nodes of $G_{cons}$, not already differentiated by previous row vectors in $\textbf{Z}_{t}$. It must also be delta consistent. These row vectors are generated and concatenated, forming $\textbf{Z}_{t}$, until all nodes are differentiated. Matrix $\textbf{Z}_{s}$ is constructed with the same vectors, sorted according to transitions in $G_{cons}$. An accelerated perceptron is trained, where each row of [$\textbf{Y}$;$\textbf{Z}_{s}$] is a sample to classify, and each column of $\textbf{Z}_{t}$ a set of classes. The algorithm stops if the perceptron training error reaches zero in less than *max_iter* iterations, and outputs all matrices. If the error is above zero after max_iter iterations, a new round of vectors that differentiate all nodes are generated and concatenated to $\textbf{Z}_{t}$, $\textbf{Z}_{s}$.

See Fig. 3 for a graphical representation of the main steps taken by gFTP when constructing a consistent graph (Fig. 3a) and constructing matrices $\textbf{Z}_{s}$, $\textbf{Z}_{t}$, $\mathbf {W_{y}}$ and $\textbf{W}_{r}$ (Fig. 3b).

## Results

### Algorithm performance

**Figure 4:**
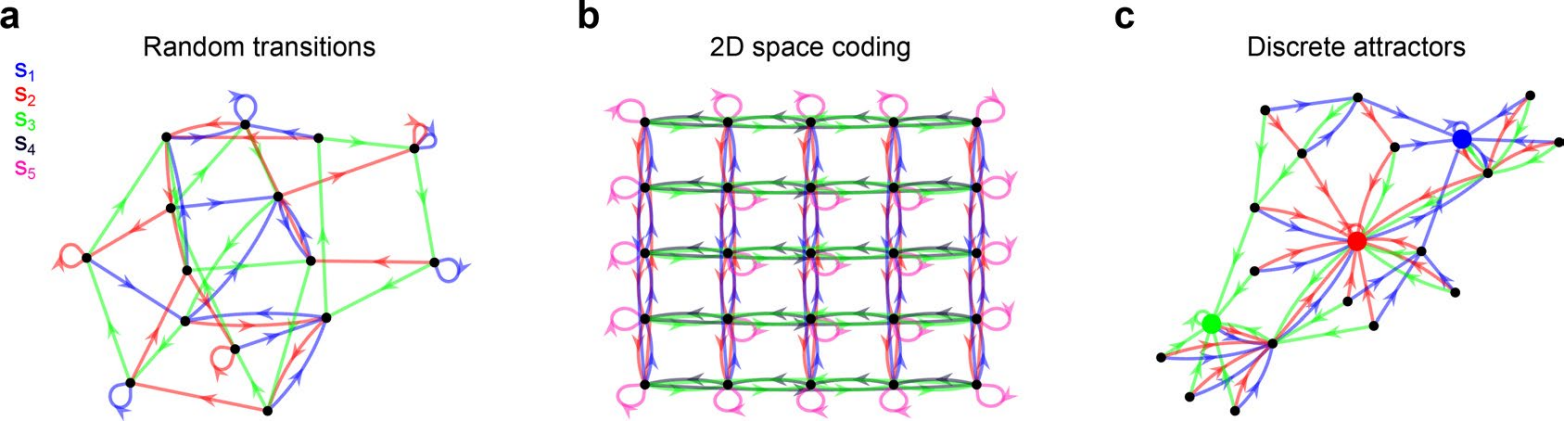
Examples of the transition graphs evaluated (before expansion) (**a**) random graph with 15 nodes and 3 stimuli. A source node *i* has target nodes taken at random from $i-2$ to $i+2$. (**b**) Transition graph of a network that encodes 25 discrete positions in a 2-dimensional square space, of side length = 5. There are five stimuli, associated with displacement to the right (green), left (black), up (blue), down (red), and a fifth stimulus (pink) associated to stillness. (**c**) Transition graph with 3 nodes acting as attractors (depicted with bigger markers). Each stimulus leads to one specific attractor through a short-distance path. Graph shown is before completing nodes without source.

We employed gFTP to construct networks from three kinds of transition graphs: random transition graphs (Fig. 4a), transition graphs that encode position in 2-dimensional space (Fig. 4b), and transition graphs that instantiate discrete attractor dynamics (Fig. 4c) (see details in “Methods”).

**Figure 5:**
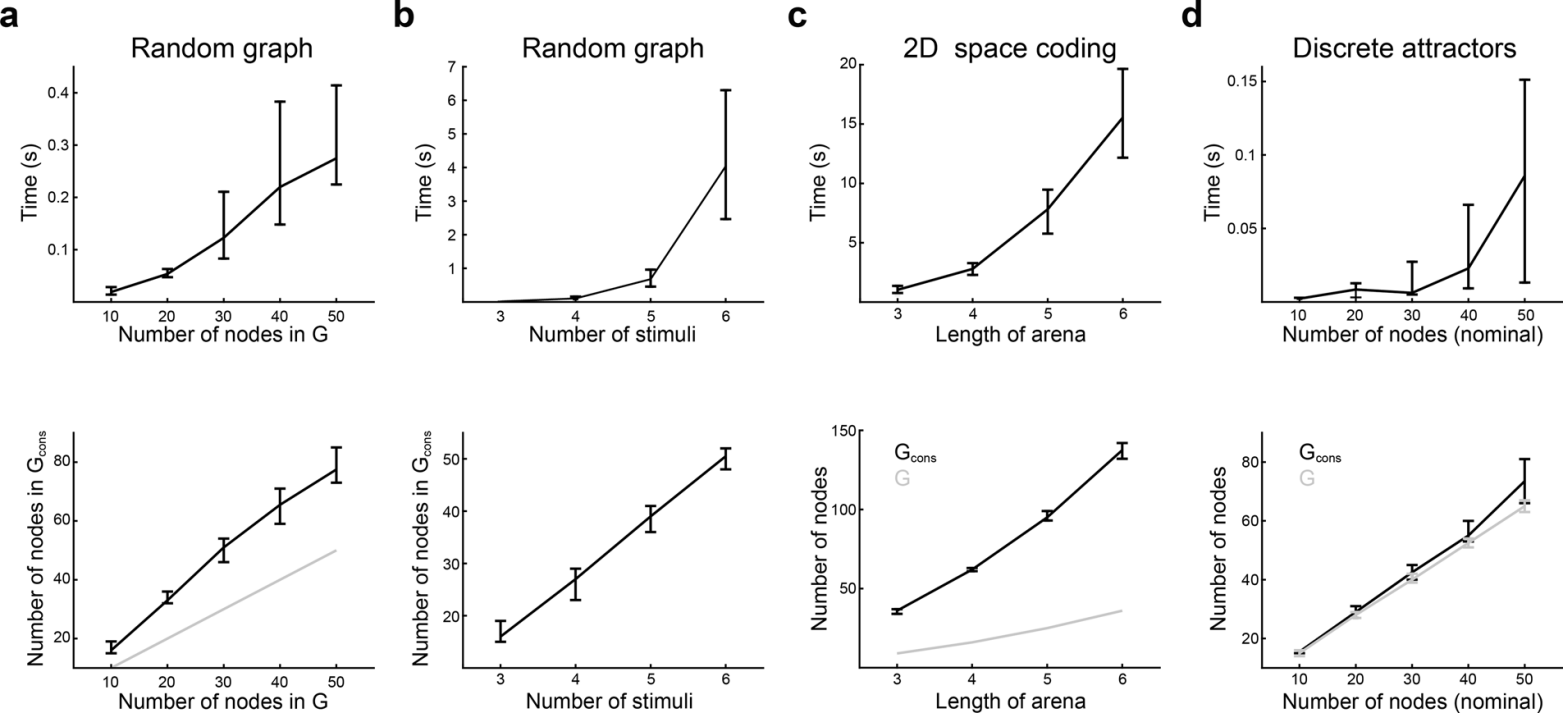
Time performance during consistency assessment. (**a–d**) Execution time (upper row), and number of nodes (lower row), until a consistent graph is obtained for the three types of transition graphs tested. *G*: initial graph, $G_{cons}$: consistent graph obtained after successive cycle detection and node expansion. (**a**) Random graphs with 3 stimuli and increasing number of nodes. The grey line is the identity function. (**b**) Random graphs with 10 nodes and increasing number of stimuli. (**c**) 2-dimensional space coding graphs, with 5 stimuli and (length of arena)$^{2}$ number of nodes (grey line). (**d**) Discrete attractor graphs, with 3 attractors, one stimulus per attractor that leads to it, and an increasing number of nodes. The *x* axis shows (nominal) number of nodes in the graph (before adding new source nodes to sourceless nodes). The *y* axis of the lower panel shows the final number of nodes in *G* after correcting for sourceless nodes, and the final number of nodes in $G_{cons}$. Median ± [25th, 75th] percentiles are shown, computed over 30 networks for each condition.

**Figure 6:**
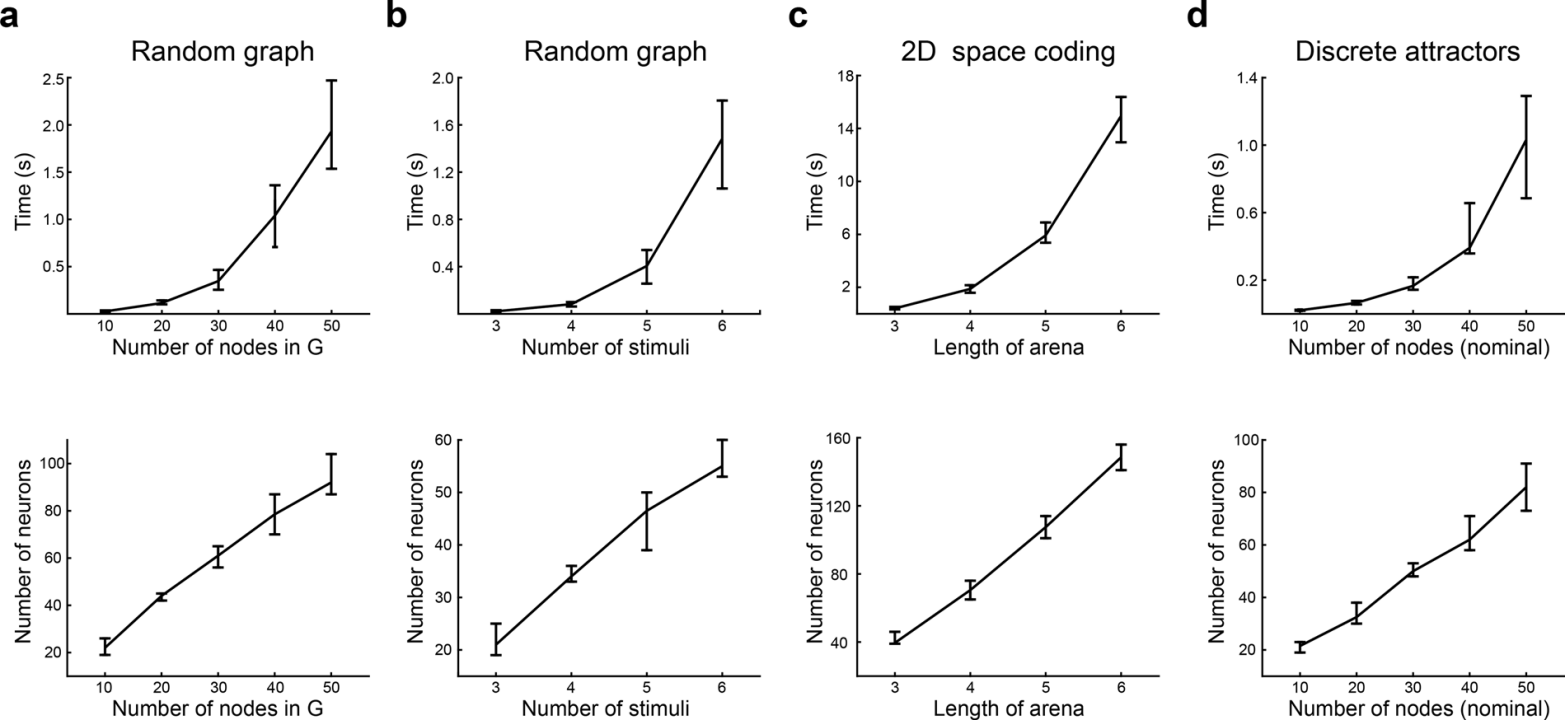
Time performance during network construction. (**a–d**) Execution times until successful construction of matrices $\textbf{Z}_{s}$, $\textbf{Z}_{t}$, $\textbf{W}_{y}$ and $\textbf{W}_{r}$ (upper row), and number of neurons in the retrieved network (lower row) for the graphs analysed in Fig. 5. Median ± [25th, 75th] percentiles are shown.

**Figure 7:**
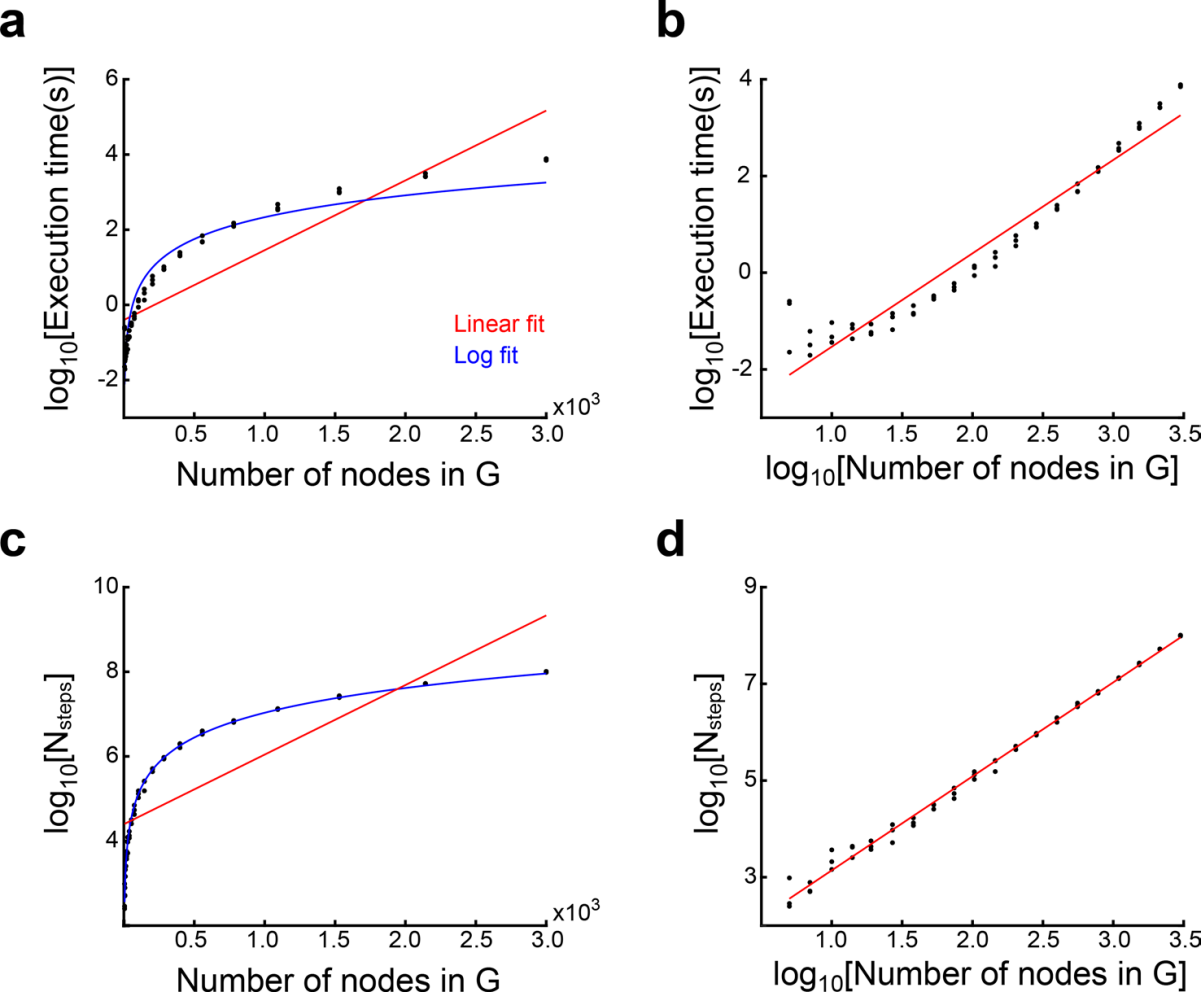
Polynomial execution time to attain delta consistency. (**a, b**) Execution time until consistency as a function of the number of nodes in *G* (before expansion). (**a**) Times are not well fitted by an exponential: linear fit $f(x)=ax+b$, $a=0.0019,\textrm{CI}=(0.0016,0.0021)$, $b=-0.40$, $\textrm{CI}=(-0.65,-0.15)$, $\mathrm {d.f.}=58$, $\mathrm {R-squared}=0.78$; log fit $a=0.84,\textrm{CI}=(0.78,0.90)$, $b=-3.5$, $\textrm{CI}=(-3.8,-3.2)$, $\mathrm {d.f.}=58$, $\mathrm {R-squared}=0.93$. (**b**) Times are well fitted by a power function: linear fit $a=1.93,\textrm{CI}=(1.80,2.07)$, $b=-3.46$, $\textrm{CI}=(-3.77,-3.15)$, $\mathrm {d.f.}=58$, $\mathrm {R-squared}=0.93$. (**c, d**) Number of executed steps ($N_{steps}$) as a function of the number of nodes ($N_{v}$) in *G* (before expansion). (**c**) Steps number are not well fitted by an exponential: linear fit $a=0.0016,\textrm{CI}=(0.013,0.0020)$, $b=4.4$, $\textrm{CI}=(4.1,4.7)$, $\mathrm {d.f.}=58$, $\mathrm {R-squared}=0.65$; log fit $a=0.84,\textrm{CI}=(0.78,0.90)$, $b=-3.5$, $\textrm{CI}=(-3.8,-3.2)$, $\mathrm {d.f.}=58$, $\mathrm {R-squared}=0.99$. (**d**) Steps number are well fitted by a power function: $a=1.9$4, $\textrm{CI}=(1.91,1.98)$, $b=1.20$, $CI=(1.11,1.28)$, $\mathrm {R-squared}=0.99$, $\mathrm {d.f}=48$. Execution times and executed steps were computed for random transition graphs, with 3 stimuli and between 5 and 3000 nodes, in logarithmic scale. Three graphs for each $N_{v}$ value were constructed.

We measured the wall-clock elapsed time during execution of the algorithm for each kind of transition graph (see “Methods” section). The complexity of the graph was adjusted by setting the number of nodes or stimuli in random graphs, the side-length of the arena in 2-dimensional space encoding graphs, or the number of nodes in the case of discrete attractor graphs. We separately measured the time required to obtain a consistent graph (consistency time, Fig. 5) and the time to construct $\textbf{Z}_{s}$, $\textbf{Z}_{t}$, $\textbf{W}_{y}$, $\textbf{W}_{r}$ matrices (construction time, Fig. 6). Wall-clock elapsed time and total number of executed steps until consistency were well fitted by a power function and not an exponential (Fig. 7), suggesting polynomial time complexity to obtain a realisable graph from a starting random graph. The number of nodes in the consistent graphs was higher than in the initial graphs (except for the discrete attractors graphs), and the relation between these two numbers was almost linear. Execution times differed considerably between graph types. Lower consistency times were found for discrete attractor graphs, followed by random graphs with 3 stimuli, random graphs with 6 stimuli, and 2-dimensional space coding graphs. Neither consistency nor construction times were fully explained by the number of nodes in the final graphs, suggesting that the shape of the graphs was an important factor in determining the number of steps necessary for making the graph consistent, and for constructing the network. The number of neurons $N_{neu}$ in the constructed network grew linearly with graph size, and was close to the number of nodes in the final graph. This implies that, in most cases, 1 neuron per node was enough, since gFTP adds neurons to the network in multiples of the number of nodes in the consistent graph.

### Robustness of the expanded transition graph

**Figure 8:**
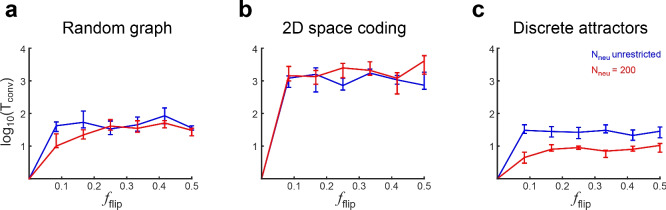
Robustness of network dynamics to initial activation state perturbations. (**a–c**) Number of iterations elapsed until a node in *Z* is reached ($T_{conv}$) plotted as a function of the fraction of neurons with flipped output at the initial activation state, for random graphs (**a**), 2D space encoding graphs (**b**), and discrete attractor graphs (**c**). Networks were constructed using gFTP, adding neurons until the accelerated perceptron found a solution ($N_{neu}$ unrestricted), or by setting $N_{neu}=200$. For this network size the accelerated perceptron found solutions without the need to add more neurons. Median ± [25th, 75th] percentiles are shown, computed on 40 networks for each $f_{flip}$ value, $N_{neu}$ condition and graph type.

Networks found by gFTP will unfold a dynamics $G_{cons}$ if the network initial activation state is one of the $\textbf{z}$ vectors in *Z*. Conversely, if this initial state departs from vectors in *Z*, we do not have any *a priori* guarantee that the network will follow $G_{cons}$. It is of interest to study how robust the network dynamics is to perturbations of its initial state. We generated transition graphs from the three analysed types, constructed the networks with gFTP, and simulated them, taking as initial activation state one activation state from *Z* and perturbing it by flipping a fraction $f_{flip}$ of the neurons’ outputs (substituting zeros with ones and vice versa). Interestingly, almost all networks eventually returned to one of the states in *Z*, therefore resuming the dynamics as specified in $G_{cons}$ (834 of 840 networks converging to $G_{cons}$, computed across all $f_{flip}$ values and graph types). In other words, most networks did not show any other stable dynamics reachable from the perturbed states, other than the dynamics specified in $G_{cons}$. This was the case for $f_{flip}$ values from 0 to 0.5. We measured how long it took networks dynamics to converge to $G_{cons}$ by computing $T_{conv}$, defined as the number of iterations elapsed from the perturbed starting point until an activation state within *Z* was reached. The $T_{conv}$ distribution was long-tailed but could be made more symmetric by taking its logarithm (Fig. 8a–c, blue line). Its value ranged from tens of iterations (for random and discrete attractor graphs, Fig. 8a, c) to the thousands (for 2-dimensional coding graphs, Fig. 8b), surpassing in many cases the number of neurons and nodes in $G_{cons}$ (random graphs: $N_{neu}=56$, $CI=[52,61]$, $N_{v}=45$, $CI=[43.5,47]$; 2D encoding graphs: $N_{neu}=66$, $CI=[60,71]$, $N_{v}=56$, $CI=[55,58]$; discrete attractor graphs: $N_{neu}=49$, $CI=[47,51]$; $N_{v}=41$, $CI=[40,42]$; median ± [25th, 75th] percentiles computed on networks across all $f_{flip}$ values).

Since gFTP stops adding neurons as soon as a solution is encountered, it could be the case that networks constructed were too small, and that adding more neurons could lead to more robust dynamics. Hence, we explored the effect that neuron number had on robustness, by constructing networks with exactly 200 neurons (3 times the biggest median network size, Fig. 8a–c, red line). In this case, convergence was faster for low values of $f_{flip}$ in the case of random graphs, and for the whole range of $f_{flip}$ in the case of discrete attractor graphs. Low $f_{flip}$ values translated in low $T_{conv}$ in some cases (the case of $f_{flip}<0.2$ and $N_{neu}=200$ in random and discrete attractor graphs), but overall the effect was small, meaning that even changing a reduced fraction of neurons resulted in a transient dynamics spanning tens to thousands of iterations (in the case of 2D attractors), until the network returns to $G_{cons}$. Therefore, networks are indeed sensitive to small changes in their initial states, but almost always converge to the dynamics they were fitted to. The sensitivity depends on the kind of dynamics, and can be reduced by increasing the neuron number, at least for random and discrete attractors graphs.

### Uncovering general dependencies between structure and function

We employed gFTP as a tool to study the multiple dependencies between neural dynamics and its underlying connectivity. To that end we implemented a genetic algorithm (GA)^31^ to optimize several measures computed on the transition graph and the synaptic weight matrix. We considered commonly employed graph theoretical measures to characterize the transition graph: the number of nodes $N_{v}$, the clustering coefficient (*c*), the modularity (*Q*) and the mutual information between stimuli and their target nodes (*I*). For these measures, we considered all arcs in the graph, regardless of their labels. To characterize recurrent synaptic weight matrix we considered: the number of neurons in the network ($N_{neu}$), the reciprocity and absolute reciprocity (*r*, $r_{abs}$), which measure the correlation between the two synaptic weights that connect each pair of neurons, and the standard deviation of out-strength ($\sigma _{out}$), a generalization for weighted graphs of the variability in out-degree distribution (see “Measures computed over transition graphs and synaptic weight matrices” in the “Methods” section, for the formal definitions).

**Figure 9:**
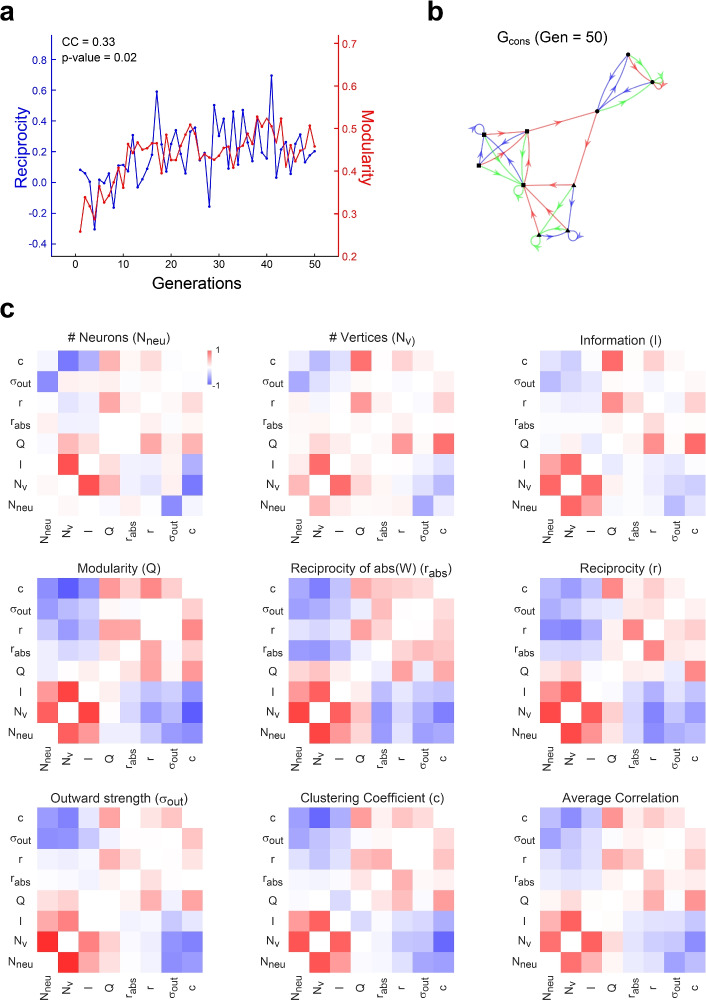
Correlational structure of dynamics and connectivity features in optimised networks. (**a**) An example of the evolutionary process. Synaptic weights reciprocity (*r*) and transition graph modularity (*Q*) are shown, computed for the elite individual in each generation of a 50 generations evolutionary process. Modularity was the fitness function in this case. Pearson correlation coefficient (CC) between both measures is shown, together with its p-value. (**(b)**) Transition graph $G_{cons}$ obtained with gFTP from the elite individual in the last generation of the evolutionary process shown in (**a**). Nodes plotted with different shapes (circle, square, triangle) indicate the three modules that maximized modularity. $Q=0.46$, $r=0.19$ for this graph and its associated network, respectively. (**c**) Correlation matrix between 8 measures that quantify distinct aspects of network dynamics and connectivity, obtained from 20 independent repetitions of the evolutionary processes. Measures computed were: neuron number ($N_{neu}$), node number ($N_{v}$), information between network state and stimulus (*I*), modularity (*Q*) and clustering coefficient (*c*) of the transition graph, reciprocity (*r*), absolute reciprocity ($r_{abs}$) and outward strength variability ($\sigma _{out}$) of the synaptic weight matrix (see “Methods” for details on each measure). Measures were always computed on the elite individual, obtaining one matrix for each repetition and for each fitness function. Each panel shows the correlation matrix, averaged across repetitions, for the fitness function indicated in the panel title. The matrix shown in the last panel is the average over all the other matrices. Colour bar in first panel shows colour scale for all panels (pure red for CC = 1, and pure blue for CC = − 1).

**Figure 10:**
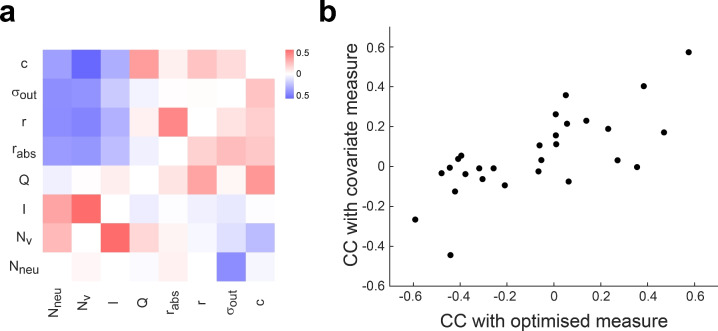
Dependence of correlation structure on the optimised measure. (**a**) Matrix showing correlations between measures along the evolutionary process. Row labels indicate the measure employed as fitness function, and collect correlation coefficients (CC) between the optimised measure and the other measures. A symmetric matrix would imply that correlations are completely independent of which measure of the correlated pair of measures was the optimised one. (**b**) Entry (*row*, *col*) in matrix shown in (**a**), plotted in the *x* axis, against entry (*col*, *row*) in the *y* axis. Spearman correlation $\rho =0.69$, $p=8.10^{-5}$. A symmetric matrix in (**a**) would result in points lying in a line of slope = 1.

We defined a population of transition graphs and a mutation function that introduced variability by permuting two target nodes, or replacing one target node with another. We ran several experiments, which differed in which measure was maximized, although all measures were computed. Then, we computed the Pearson correlation coefficient (CC) between each pair of measures along the evolutionary process (e.g. Fig. 9a, b). In this way we expected to uncover how constraining (maximizing) one feature, either related to network dynamics or connectivity, impacts on the correlational structure of all other measures. On average, measures could be grouped into two clusters, each defined by positive intra-cluster and negative inter-cluster CC (Fig. 9c): Cluster 1, composed of $N_{v}$, $N_{neu}$, *I*, and *Q* (modularity), and Cluster 2, composed of *r*, $r_{abs}$, $\sigma _{out}$, and the *c*. The positive CC between $N_{neu}$ and $N_{v}$ in Cluster 1 is expected, since more neurons are required to generate enough population states. Also, the positive CC between $N_{v}$ and *I* can be explained as an effect exerted by nodes added during graph expansion: new nodes only encode one stimulus when they are first added to the graph, thus contributing to a higher mutual information between node and stimulus. In Cluster 2, the positive CC between modularity and the clustering coefficient was also expected. Modularity was the only measure that showed a tendency for positive CC in most cases, even when the clustering coefficient showed negative CC. Reciprocity and absolute reciprocity were positively correlated with modularity and clustering coefficient, revealing an interesting relationship between a structural feature and a dynamical feature. Higher than chance reciprocity has been found experimentally^32^, and it has been connected to optimal memory storage^33^ and stimuli sequence encoding^18^. Results in Fig. 9 suggest that it may also subserve modular dynamics in general, which in turn can be related to specialization of function. The correlations analysed so far are clearly distinguished in the mean correlation matrix. A similar tendency can be recognized in the correlation matrix computed separately for each optimised measure, albeit with some departures from the average. To assess the immediate impact that an optimised measure has upon the others, we computed a correlation between two vectors of CCc: one vector collects the CCs between measure *i* and measure *j*, when measure *i* was optimised; the other vector collects the CCs between measure *i* and measure *j*, when measure *j* was optimised. These two vectors were positively correlated (Fig. 10a, b), meaning that there is a tendency to find the same dependencies between measures regardless of which one was optimised. Even so, the unexplained variance was considerable. All in all, this suggests that the correlational structure between dynamical and structural measures has one component, which does not depend on the optimization process, and another component, which may depend on which measure is optimised, and even the entire optimization history.

### Assessing dependencies between dynamics, connectivity, and the algorithm instantiated by the network

**Figure 11:**
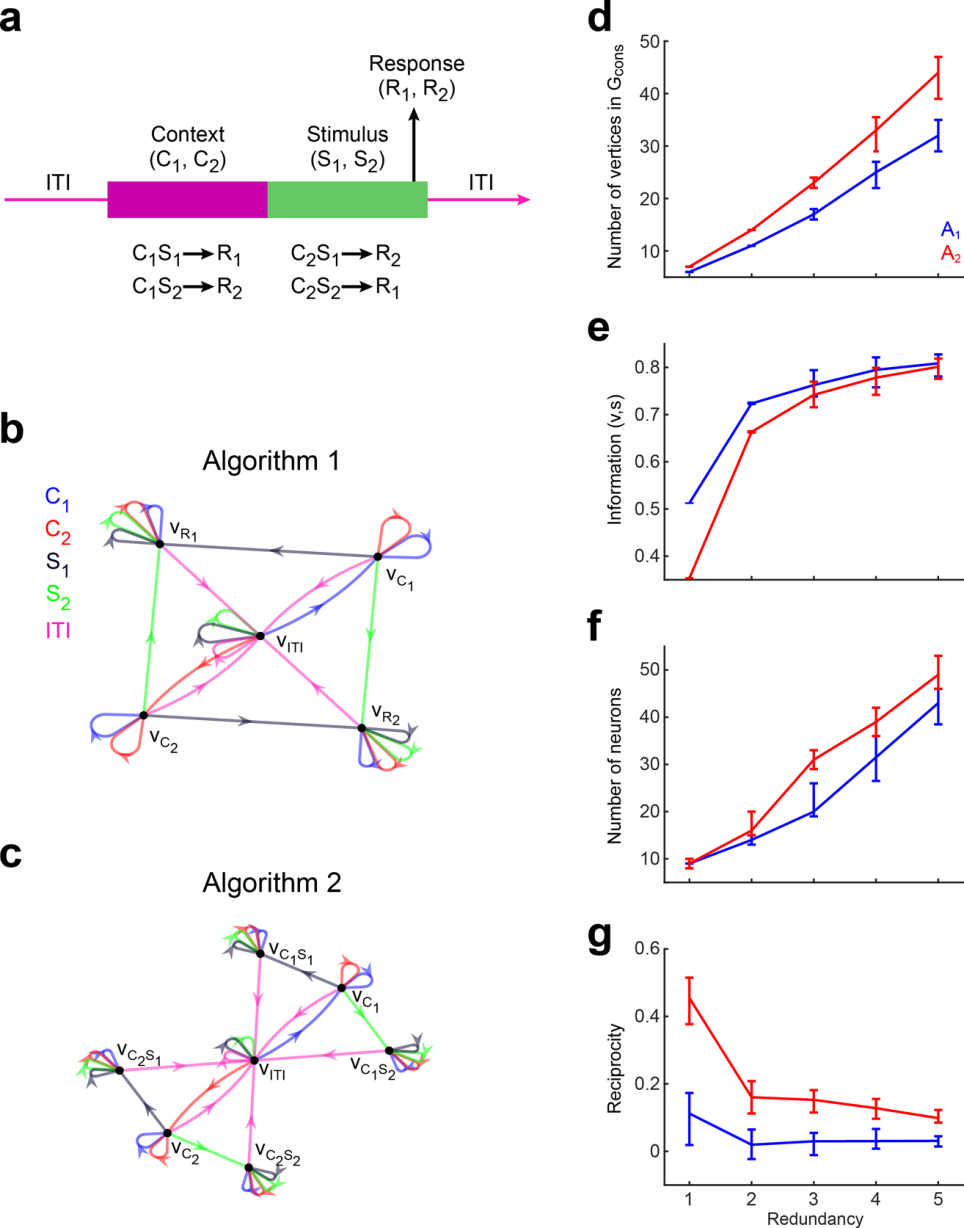
Effect exerted by the instantiated algorithm on the network dynamics and connectivity. (**a**) Context-dependent discrimination task. A trial starts with the presentation of a context cue, indicating the correct stimulus-response mapping for the trial, followed by the stimulus to discriminate. The agent is expected to execute the correct response after stimulus presentation. (**b**) Transition graph for Algorithm 1 (A1). Nodes $v_{R_{1}}$ and $v_{R_{2}}$ encode the correct response, which depends on context and stimulus. Once the system is in one if these states, it is impossible to recover the stimulus and context that led to the decision. **(c)** Transition graph for Algorithm 2 (A2). Similar to A1, but it has nodes $v_{c_{1}s_{1}}$, $v_{c_{1}s_{2}}$, $v_{c_{2}s_{1}}$, $v_{c_{2}s_{2}}$, which simultaneously encode stimulus and context, and therefore convey the necessary information to trigger the correct response ($R_{1}$ with $v_{c_{1}s_{1}}$ and $v_{c_{2}s_{2}}$, and $R_{2}$ with $v_{c_{1}s_{2}}$ and $v_{c_{2}s_{1}}$). Self loops render states refractory to stimuli that are out of place, like a context cue during stimulus presentation, or a second presentation of a stimulus when the stimulus was already presented and encoded. (**d–g**) Quantification of differences in dynamics and connectivity features between networks instantiating A1 or A2, with increasing levels of redundancy. Redundancy shown in the x axis indicates how many times the number of nodes in graphs shown in (**c**, **d**) was multiplied by adding redundant nodes. Measures were computed on the $G_{cons}$ and synaptic weight matrices obtained through gFTP. Median ± [25th, 75th] percentiles are shown, computed on 100 graphs and their respective weight matrices, per redundancy level.

We sought to understand how two ways of solving the same task (two different algorithms) constrain the connectivity and dynamics of the network that solves it. To that end, we considered a stimulus discrimination task with two possible stimulus-response mapping, indicated by a context cue (Fig. 11a). The cue is presented first, followed by the stimulus to discriminate. Once the stimulus is presented, the network has all the information to execute the correct response, and therefore all information encoded in the neural population regarding context and stimulus can be discarded. We call this case Algorithm 1 (A1, Fig. 11b). Nevertheless, context and stimulus information could be retained, for purposes other than solving the task at hand, as has been observed experimentally^34^. This is Algorithm 2 (A2, Fig. 11c). We constructed networks for each algorithm, and measured the number of nodes in the consistent graph, mutual information between stimulus and node, the number of neurons in the constructed network, and its reciprocity. We also assessed the impact of redundancy in network states. Redundancy was accomplished by expanding the initial transition graph with new nodes that encoded no more information than the original nodes (similar to expansion when constructing a consistent graph, see “Methods”). Algorithms 1 and 2 could be sorted out based on these measures, despite the small differences between their transition graphs (Fig. 11d–g). Higher redundancy led to higher $N_{v}$ and *I* in the consistent graphs, and higher number of neurons and lower reciprocity in the networks, for both algorithms. This is consistent with results in Fig. 9. However, with matching redundancy levels, Algorithm 1 showed lower $N_{v}$ than Algorithm 2, but higher *I*, contrary to the positive correlation found in Fig. 9. Networks executing Algorithm 1 had fewer neurons than networks executing Algorithm 2, and less reciprocity, which is also in opposition to the negative correlation these two measures exhibited in Fig. 9. If these results generalise, they would suggest that lower *I* and higher reciprocity are among the distinctive traits of networks that instantiate more complex algorithms. They would also imply that the dynamics and connectivity measures here considered depart from the main dependencies observed in Fig. 9 when specific transition graphs are analysed.

## Discussion

We have introduced gFTP, an algorithm that takes a target dynamics as input and returns the connectivity matrix of a neural network that follows the specified dynamics. gFTP detects if the target dynamics, described in terms of a transition graph, is not realisable by a neural network, in which case the transition graph is expanded until a realisable transition graph is reached, one that encodes all the information conveyed in the original graph. Realizability is attained by transforming a problem of linear constraints into a cycle detection problem, in which the presence of a cycle indicates an inconsistency, and the absence of cycles means that a realisable graph has been found. This allows us to find consistent transition graphs that are more compact (have fewer nodes) than previous methods in which the expanded graph had as many nodes as (stimulus, node) pairs in the original graph^29^.

Our approach departs from other model building approaches in several ways. In bottom up approaches, a model is a specific formalization of a series of proposed mechanisms. Its dynamics is a complex emergent of these mechanisms, and in most cases it cannot be specified in advance. Although it is possible, for some models, to prove analytically some of their properties (like in^35,36^), this is mostly done at the cost of many simplifications. Complex dynamics that can instantiate specific algorithms are difficult for analytic treatment. On the other hand, normative approaches constrain dynamics to the subsets of dynamics that minimize the loss function, but neural network models have enough degrees of freedom to find very disparate solutions. While a multiplicity of solutions is appealing in itself, as a way of exploring novel dynamics, it also makes it problematic to go from specific solutions to general conclusions regarding mechanisms. In this sense, our algorithm brings a new angle to the way we build neural networks as models of brain function, by providing a high level of control over the network dynamics. In turn, this higher control can be exploited in several ways:

1. We can, for instance, construct networks which exhibit dynamics that have been proposed theoretically, or found experimentally, like the discrete attractor dynamics proposed to subserve working memory, or the toroid dynamics proposed to subserve spatial coding. We can also construct networks whose dynamics instantiate algorithms that have been proposed to subserve some aspect of cognition or behaviour. This aspect is particularly specific to our approach, since normative methods are not well suited for specifying exactly how the network solves a task. This makes gFTP particularly useful for studying the interdependencies between dynamics and the algorithms it implements, the information encoded in the activation states, and the network connectivity. We may define different algorithms that solve the same task and then compare the expanded graphs, or the networks obtained, and draw conclusions about how the differences in the algorithms translate into differences in dynamics or network connectivity. If it happens that gFTP finds a graph to be inconsistent, then it means that the proposed dynamics cannot, as such, be realisable in a neural network. This fact is in itself useful, because we can study which features of the dynamics are essential to a given algorithm, and what features are there because of the constraints imposed by the fact that dynamics has to be carried out by the neuronal machinery.

2. For each transition graph that is inconsistent there are many different ways to expand it into a consistent one. We can then run gFTP multiple times, sampling from the distribution of expanded graphs that come from the same initial graph, and study how different the expanded graphs can be.

3. Once we have a consistent graph, we can generate activation states in a way that they all share some target activation statistics (e.g. a given mean population firing rate and pairwise correlation). Then, we can assess how the dynamics/algorithms constrain those statistics.

4. For each dynamics and each set of activation states, we can generate samples of synaptic matrices that instantiate the same dynamics with the same activation states, and assess how dynamics and firing statistics impact on network connectivity.

In sum, gFTP provides a high level of control over key aspects of analysis, like network function, dynamics and firing statistics, allowing to disentangle their interactions by carrying out controlled numerical experiments. Comparisons between gFTP-generated models and neurophysiological data can be conducted at the level of firing rate statistics, such as mean firing rates or pairwise correlations. Additionally, models and neural recordings can be related at the level of the topological properties of their latent spaces, obtained through dimensionality reduction techniques^15^. In contrast to system identification approaches, which emphasise quantitative fits to neural data through statistical methods^52^, gFTP is best posited to study qualitative relationships between network structure and function.

Our results with random graphs showed a time complexity well fitted by a power function of the number of nodes, with exponent ~1.94, for the operations of consistency assessment and expansion. This probably stems from the fact that the main subroutine for these operations is Depth First Search, which has linear time complexity on the number of nodes and arcs (which becomes quadratic at most, in the number of nodes, in the case of a fully connected graph). On the other hand, to construct $\textbf{Z}_{t}$ our current implementation explores the tree of possible assignments for each neuron in each network state, accelerating the process by computing deltas and filling assignments that are completely specified by the deltas and *z* values already defined. We also used the heuristic of assigning *z* value first to nodes with higher degree in graph *G*, for these are expected to be part of cycles with higher probability. This heuristic helped in reducing execution time during construction of matrices $\textbf{Z}_{t}$. Yet, the algorithm would benefit from other ways of assigning *z* values. Studying the structure of graph *D* and cycle formation will certainly help. Also, optimization methods like genetic algorithms could be good options to evaluate.

Once matrices $\textbf{Z}_{t}$ were constructed, we found matrices $\textbf{W}_{y}$, $\textbf{W}_{r}$ by employing the accelerated perceptron algorithm. We decided to add neurons to the network in multiples of the number of nodes in the transition graph. The reason for this criterion is that, for matrices $\textbf{W}_{y}$, $\textbf{W}_{r}$ to exist, matrix $\textbf{U}$ must have the same linear combinations that matrix $\textbf{C}$, and $\mathcal{H}(\textbf{U})=\textbf{Z}_{t}$. Not all matrices $\textbf{Z}_{s}$ and $\textbf{Z}_{t}$ allow the existence of a matrix $\textbf{U}$ that fulfils these two conditions. Yet, the probability of finding one viable matrix $\textbf{U}$ is expected to be higher if the number of linear combinations in $\textbf{C}$ is low. This can be achieved by adding neurons to the network, with random values of *z*, since the probability of a linear combination between rows of $\textbf{Z}_{s}$ is expected to drop with the number of its columns. Adding neurons in multiples is also convenient because the number of calls to the accelerated perceptron is reduced: too few neurons would certainly not lead to a solvable system, and the accelerated perceptron will have to reach the maximum number of allowed iterations in order to stop and try with a higher number of neurons, thus increasing execution time. In our experiments, a number of neurons approximately matching the number of nodes was enough to define a solvable system of linear separation problems.

Networks composed of binary threshold units have a long history as models of brain function. Despite their simplicity, they have been successfully employed to model relevant aspects of brain dynamics and computation^33,37–41,53^. We think that there are still many yet-to-be-explained phenomena that can be studied with these kind of networks. Most models cited above were the result of optimisation, or were constructed by hand. We thus believe that the capabilities offered by gFTP, in terms of control of network dynamics and the possibility of tinkering with the network state statistics, will open new opportunities for applying these simple models to uncover properties of biological neural circuits, through the exploration of more complex networks, built upon a richer set of hypothesis regarding the algorithms they instantiate. On the other hand, gFTP in its current form is restricted to networks of binary neurons, because the way the algorithm constructs a realisable transition graph has, so far, no obvious extrapolation to other neuron models. The feasibility of such extrapolation is an interesting challenge for future work. Another limitation of our method is that, for constructing matrix $\textbf{Z}_{t}$, we rely on a backtracking approach, which can be inefficient. Better methods could be developed in the future for this task, as we have discussed above. Also, matrix $\textbf{Z}_{t}$ could be generated only to be close to consistent, and $\textbf{W}$ only leading to an approximation of $\textbf{Z}_{t}$. In this case, the obtained network will not follow the target dynamics exactly. However, it is expected that, with enough neurons and redundancy in $\textbf{Z}_{t}$, the approximate network will follow a dynamics that is close enough to the target. It could also be possible to use the network found with gFTP as a good starting point for further optimization with another algorithm.

The gFTP algorithm is suitable for studying the interdependence between network parameters and its function, by generating a sample of networks that share a given target property. Recent works have addressed similar goals. In Brennan *et al*.^42^ a method termed LOOPER was introduced, in which a Markov process is constructed from recorded neural activity, such that the global dynamics respects convergences and divergences observed in the data. The method relates to ours in that it allows to construct a model of an arbitrary dynamical system. LOOPER has the advantage of finding a model directly from data, while the use of a binary neuron model in gFTP makes it more difficult to translate recorded firing activity into a transition graph. However, and in contrast to our method, LOOPER never constructs a proper neural network model, so there is no explicit way of linking the low dimensional dynamics recovered in the model with any underlying network connectivity. On the other hand, in the work of Brittner et al.^43^ and Gonçalvez et al.^44^ a deep neural network was employed to approximate a probability density function over parameters of a target neural network, such that a target mean over an emergent property is satisfied. Gonçalvez method has the added benefit of allowing non-differentiable models. The computational cost of both methods is an issue, requiring several hours for networks of up to 1000 parameters. Conversely, although gFTP generates one network at a time, each generation is fast, hence a big population of networks can be gathered for subsequent analysis.

In conclusion, we have introduced a systematic way of constructing neural networks of binary neurons that unfold a user-specified dynamics. Its efficiency and versatility make gFTP a powerful tool for exploring new hypotheses regarding the connectivity and dynamics underlying brain function. We believe the gFTP algorithm is going to be a valuable addition to the toolbox of the theorist.

## Methods

### Notation

**Table 1:** Symbols with descriptions.

Symbol	Description
$N_{neu}$	Number of neurons in the network
$\textbf{z}$	Vector containing the outputs (activations) of each neuron
$\textbf{u}$	Vector of preactivations such that $\mathcal{H}(\textbf{u})=\textbf{z}$
$\textbf{W}_y$	Matrix of synaptic weights between sensory inputs and neurons in the network. The ith row collects incoming weights from sensory inputs to the ith. neuron
$\textbf{W}_{r}$	Matrix of synaptic weights between pairs of neurons in the network. The ith row collects incoming weights from all neurons to the ith neuron
*G*	Labeled multidigraph with one node per network state and one arc for each transition. Arcs are labeled by the stimulus that triggers the transition
$N_{v}$	Number of nodes in *G*
$N_{tran}$	Number of transitions (arcs) in *G*
*v*	A node in *G*
*V*	The set of all nodes in *G*
$f_{s}$	The function that maps arcs in *G* to their source nodes
$f_{t}$	The function that maps arcs in *G* to their target nodes
$l_{G}$	The function that maps arcs in *G* to their labels
$\textbf{G}$	Matrix representation of graph *G*, with one row for transition. Columns contain stimulus, source node, and target node
$\textbf{Y}$	Matrix of input vectors. The ith column collects the input vector $\textbf{y}$ associated with the stimulus that triggers the ith transition in *G* (the ith row in $\textbf{G}$)
*Z*	The set of network activation states $\textbf{z}$. It maps to *V* in a one-to-one fashion
$\textbf{Z}_{s}$	Matrix of activation states, when acting as sources in *G*. The ith column collects the activation states associated with the source node of the ith transition/row in $\textbf{G}$
$\textbf{Z}_{t}$	Matrix of activation states, when acting as targets in *G*. The ith column collects the activation states associated with the target node of the ith transition/row in $\textbf{G}$
$\textbf{U}$	Matrix of preactivations, such that $\mathcal{H}(\textbf{U})=\textbf{Z}_{t}$
$\varvec{\delta }_{i,j}$	Equal to $\textbf{W}_{y}(\textbf{y}_{s_{i}}-\textbf{y}_{s_{j}})$. Its length is $N_{neu}$. It collects the difference between contributions of stimulus $s_{i}$ and $s_{j}$ to preactivations reached after any transition
*D*	Labeled multidigraph. Its nodes are contained in *V*. Two nodes are connected if they are reached through stimuli $s_{i}$ and $s_{j}$ from a common source node in *G*. Arcs are labeled by $\delta _{i,j}$
$g_{s}$	Function that maps arcs in *D* to their source nodes
$g_{t}$	Function that maps arcs in *D* to their target nodes
$l_{D}$	Function that maps arcs in *D* to their source labels
*C*	The set of all labels/deltas in *D*
*P*	A partition of *C*
$V_{trav}(C_{i})$	A set that collects (source, target) node pairs of arcs in *D*, whos labels are in element $C_{i}$ of partition *P*

### Constructing matrices $\textbf{Z}_{s}$ and $\textbf{Z}_{t}$ with backtracking

To ensure that all nodes are differentiated, we define neurons so that their output is different for at least a pair of nodes. Thus, we choose two nodes ($v_{1}$ and $v_{2}$) that are going to be differentiated. First, the algorithm assigns value 1 to node $v_{1}$ and 0 to $v_{2}$. Next, values 1 or 0 are assigned to each one of the remaining nodes, one at a time, following a list $L_{u}$ of undefined nodes sorted in decreasing in-degree order. The first element in this list is set as the active element, the one that is going to be defined. Any node that is set as active is registered in a list of active elements $L_{a}$. Each new assignment may define a new delta, so delta consistency must by checked. If consistency is sustained, new node assignments are made as necessary, according to the *z* and delta values defined so far (propagation of values according to delta consistency). Then, consistency is checked again. If sustained, the first non defined node in $L_{u}$ is set as the active element. The process continues with the new active element until all nodes have *z* value. If delta consistency is violated, another *z* value is tried for the active element (1 if 0 was already tried, 0 if 1 was already tried). If neither of the assignments was successful, the algorithm takes a step back by searching for the last successfully assigned active element in $L_{a}$, making it active, and erasing from the list all elements after it. All nodes that were defined after the active element are set as undefined. Assigned values are removed from the list of viable values for that node, but all values are restored after a step back, for all the following nodes in $L_{u}$, after the active one. If all *z* values were tried for the first node in $L_{u}$, without success, then the algorithm stops with no $\textbf{z}$ as output (there is no node left to step back), and hence the transition graph is delta inconsistent. A graph that was previously expanded to obtain a consistent graph $G_{cons}$ always leads to a fully assigned $\textbf{z}$ for any pair of differentiated nodes.

Once we get vector $\textbf{z}$, we add it as a row vector to $\textbf{Z}_{t}$, and identify all nodes that were differentiated (which can be more than just $v_{1}$ and $v_{2}$), removing them from the list of pairs of nodes to differentiate. If all pairs of nodes were already differentiated, then each node has one vector $\textbf{z}_{v}$ that encodes that node. At this point we can construct $\textbf{Z}_{s}$ . Matrices $\textbf{C}=(\textbf{Y};\textbf{Z}_{s})$ and $\textbf{Z}_{t}$ define $N_{neu}$ linear separation problems (one for each neuron), where the $N_{tran}$ column vectors in $\textbf{C}$ are the points to separate, and the $N_{tran}$ ones and zeros of each row in $\textbf{Z}_{t}$ are the classes. For each neuron we find its input weights by fitting a perceptron through the accelerated perceptron learning algorithm (algorithm No. 2 in^30^). If after *max_iter* learning iteration one or more neurons are encountered for which the separation problem cannot be perfectly solved, we assume that at least one linear combination in $\textbf{Z}_{s}$ cannot be reproduced in $\textbf{U}$. In this case, new matrices $\textbf{Z}_{s}$ and $\textbf{Z}_{t}$ are generated and concatenated to the previous ones, to increase the number of neurons and reduce the number of linear combinations in $\textbf{Z}_{s}$. This process is repeated until each neuron can be perfectly solved. It is known that the probability of finding a linearly separable problem increases with the dimensionality of the input vector (the number of neurons in our case) for a constant number of patterns to separate^15,45^. Moreover, the perceptron algorithm is guaranteed to converge to a solution in a finite number of steps, provided such a solution exists^46^. Therefore, since gFTP adds neurons but the number of transitions is constant, there will be a finite number of neurons for which all linear separation problems can be solved.

In practice, we frequently observed cases where all pairs of nodes were differentiated but the accelerated perceptron failed to find a solution, meaning that more neurons were required. Therefore, each round of $\textbf{Z}_{s}$ and $\textbf{Z}_{t}$ matrices that are concatenated to the previous ones were constructed by adding neurons until all pairs of nodes were differentiated *and *the total number of neurons surpassed the number of nodes in $G_{cons}$.

### Transition graphs analysed

*Random transition graphs*: We set a number of nodes and stimuli. For each node, we defined its target nodes reached through each stimulus. Nodes were numbered, and target nodes were taken at random, with replacement, from the list of nodes that went from 2 nodes before the source node, to 2 nodes after the source node, avoiding self-targeting. Therefore, transitions were at random, but constrained to a vicinity around the source.

*2-dimensional continuous-like attractor*: We defined a discrete 2-dimensional space, square in shape, of a certain integer side length. There was one node for each discrete position in this space (i.e. 9 positions and nodes for a 3 $\times$ 3 square arena). Parallel sides were glued together, forming a torus. We defined 5 stimuli, 4 for vertical or horizontal displacements, and a 5th for stillness. Therefore, the transition graph instantiated a discretised version of the dynamics of a continuous attractor model, thoroughly studied in the context of spatial coding^16^.

*Discrete attractors*: we defined a number of nodes, and a number of stimuli, which was the same as the number of discrete attractors (each stimulus had an associated attractor node, and that stimulus led to its associated attractor). Then, we assigned to each node a random position in a 2-dimensional space, and defined a graph in which each node was connected to its closest 6 nodes, measured in Euclidean distance between positions. Finally, we found the shortest path from each node to each of the attractor nodes. We constructed graph *G* by making each stimulus trigger a transition to the first node in the shortest path pointing to the attractor associated with that stimulus. Since some nodes could end up being sources without being targets, for each one of these sourceless nodes we introduced one extra node, that went to the sourceless node through one stimulus, and to itself through the remaining stimuli.

*Context dependent discrimination*: We considered a task in which the agent must choose one out of two possible responses when presented with one out of two possible stimuli. There were two rules (or mappings) by which stimulus and response might be matched: ($S_{1}$, $R_{1}$), ($S_{2}$. $R_{2}$), or ($S_{1}$, $R_{2}$) ($S_{2}$, $R_{1}$). In each trial the rule is chosen at random, and indicated through a context cue ($C_{1}$ or $C_{2}$), which is presented immediately before the stimulus to discriminate. The response is observed after stimulus presentation. Trials are separated by an inter-trial interval (ITI). We considered a recurrent neural network that receives the context cue and stimulus as input. It also receives a specific input that represents the ITI, and puts the network into a basal state. The context cue pushes the network to a context coding state ($v_{C_{1}}$ and $v_{C_{2}}$). From this state, the network is sensitive to the stimulus, which triggers a transition to the correct response coding state. We did not model a dedicated network to execute the response, and instead we only cared that the correct response was decodable from the population state. We considered two possible algorithms for this task. In Algorithm 1 stimuli trigger transitions from $v_{C}$ to the population state that encodes the correct response. This state can be reached through stimulus $S_{1}$ or $S_{2}$, depending on context, implying that states $v_{R_{1}}$ and $v_{R_{2}}$ do not encode context nor stimulus per se. Therefore, the information regarding context and stimulus is lost when the response is executed. On the contrary, in Algorithm 2, the population state that encodes the correct response is different depending on the context state and the stimulus presented. This causes the states that encode the correct response to also encode the context and the stimulus presented. All the information is preserved.

### Empirical assessment of execution time complexity

We measured the wall-clock execution time by means of the tic toc MATLAB commands. Consistency times were obtained by measuring execution times of the Make_Consistent function (first line in Algorithm 1 in Supplementary Information). Construction times were obtained by measuring the execution times of lines 2–13 of the same algorithm.

To measure the execution times shown in Fig. 6 we generated random graphs of 5–3000 nodes, making exponentially increasing steps such that the node number increases linearly on a logarithmic scale. Next, we measured the execution time of the Make_Consistent function.

To have an estimate of time complexity in terms of number of operations of the Make_Consistent function (Algorithm 2 in Supplementary Information), we counted the total number of steps required for the function to reach a consistent graph. We added one step to the step counter for each *for* loop and *while* loop executed by our MATLAB implementation of the algorithm. This included all *for* loops in calls to Make_D, all cycles in DFS, each time Make_Consistent reaches line 15, each time Find_Superposition (Algorithm 3) reaches lines 10 and 16, and each time Expand_Node (Algorithm 5) reaches lines 6 and 10.

### Robustness assessment

We simulated the networks obtained through gFTP, taking a random $\textbf{z}$ from *Z* as initial state, and perturbing it by changing a fraction $f_{flip}$ of neuron activations, replacing zeros for ones and vice versa. We executed the network during $N_{iter}=10^{3}N_{v}$ iterations, with $N_{v}$ being the number of nodes in $G_{cons}$. Then, we computed $T_{conv}$ as the number of iterations elapsed from the initial state until the first occurrence of a network activation that is a member of *Z*. If no activation state was equal to any of the activation states in *Z*, the network was excluded from the analysis of $T_{conv}$ shown in Fig. 8, but counted as a non-convergent network for the number of convergent networks reported in the “Results” section.

### Optimisation with a genetic algorithm

We constructed a population of 30 individuals. Each individual is a transition graph *G*, possibly not realisable, with $N_{v}=5$ and number of stimuli $N_{s}=3$. For each *G* a graph $G_{cons}$ is constructed with gFTP, together with the synaptic weights of the network that instantiates $G_{cons}$. Next, the fitness of each individual is computed, by applying the fitness function to the $G_{cons}$ or synaptic weight matrix of that individual. The fitness function is chosen from a set of measures, listed below. Then, individuals are selected with replacement with probability proportional to their fitness. A mutation operator introduces variability to each selected individual by applying one of two equally likely modifications to *G*: permutation of the target node of two different transitions, or replacement of the target node of a transition by another node. In the last case, only nodes that appear more than once as targets are considered to be replaced, to avoid the disappearance of any node. Therefore, all graphs will remain having $N_{v}$ nodes throughout the evolutionary process. The new population is composed of the mutated individuals, plus an unaltered copy of the best individual in the last generation (called the elite individual). We were interested in finding how maximising a given feature of the transition graph or the synaptic weight matrix affects other dynamical or connectivity features. To do this, we computed a series of measures on the elite, and kept track of them along the evolutionary process. To obtain the data summarised in Fig. 9, we conducted the evolutionary process 20 times, during 50 generations each, computed correlations between the tracked measures, and averaged them over the 20 repetitions.

### Measures computed over transition graphs and synaptic weight matrices

The measures analysed and selected as fitness functions were:

- $N_{v}$: the number of nodes in the consistent graph obtained through gFTP.
- Clustering coefficient (*c*): Defined as the average computed over all per-node clustering coefficients. The clustering coefficient of a node is the number of triangles the node is part of, divided by the number of all possible triangles there could be for that node^47^.
- Modularity (*Q*): the number of arcs within modules minus the expected number of arcs in a random graph with matching degree distribution. We employed the Leicht and Newman algorithm^48^ to partition nodes into modules that maximise *Q*.
- *I*: the average information encoded in a network state about the stimulus that leads to it. Formally, the average normalized mutual information between a node taken as target and the stimuli that lead to it:
  12 $$\begin{aligned} I&=\frac{1}{N_{v}}\sum _{i=1}^{N_{v}}1-\frac{H_{v_{i}}}{H_{max}} \end{aligned}$$
  13 $$\begin{aligned} H_{v}&=-\sum _{j=1}^{N_{s}}p_{v}(j)\,\textrm{log}_{2}(p_{v}(j)) \end{aligned}$$
  14 $$\begin{aligned} p_{v}(j)&=\frac{n_{s_{j},v}}{n_{v}} \end{aligned}$$
  with $N_{s}$ the number of stimuli, $H_{max}=\mathrm {log_{2} N_{s} }$, $n_{s_{j},v}$ the number of arcs with label $s_{j}$ that target *v*, and $n_{v}$ the total number of arcs that target *v*.
- $N_{neu}$: the number of neurons in the recurrent neural network.
- Reciprocity (*r*): The Spearman correlation coefficient computed between a vector collecting the weights from neuron *i* to neuron *j*, and a vector collecting the matching weights from neuron *j* to neuron *i*.
- Absolute reciprocity ($r_{abs}$): Reciprocity computed over the unsigned recurrent synaptic weights.
- Standard deviation of out-strength ($\sigma _{out}$): measures the variability on the outward synaptic weights. We compute, for each node, the outward strength, defined as the mean over outward weights. Then, we computed the standard deviation over the outward strengths of all nodes.

The clustering coefficient and modularity were computed with the Brain Connectivity Toolbox^49^. Graph measures included all arcs in the graph, regardless of their labels.

Before computing any synaptic weight measure, we first divided the inward weight vector of each neuron by its Euclidean norm. This transformation leaves the neuron input-output mapping intact, and reduces weight variability accumulated during the accelerated perceptron learning process.

The minimum value for reciprocity and absolute reciprocity is − 1, hence we added + 1 to these measures when used as fitness function.

### Adding redundancy to *G*

Redundant codes are a hallmark property of robust systems, neural systems included^50,51^. We introduced redundancy to a transition graph *G* by adding nodes that encode the same information as another preexisting node. Two nodes encode the same information if they are reached by the same sequence of stimuli and nodes. To accomplish this, we added new nodes, each one equivalent to a randomly chosen preexisting node, i.e. the new node leads to the same target node, and through the same stimuli, as its equivalent node. We say that equivalent nodes are members of the same equivalence set. Each node belongs to one equivalence set. There is one equivalence set for each node in the original graph, and they are mutually disjoint. Each time a new node is added, its target nodes are taken at random from a randomly chosen equivalence set. For results in Fig. 11 we added one new node for each equivalence set, and repeated this process $N_{red}$ times, with $N_{red}$ being the redundancy level.

### Supplementary Information

Supplementary Information.

## Data Availability

The gFTP MATLAB implementation used to generate all data and figures is available on a public GitHub repository: https://github.com/cmininni/gFTP.git.
