# Supplementary material for: Constructing neural networks with pre-specified dynamics
Source: Sci Rep. 2024 Aug 14;14:18860. doi: 10.1038/s41598-024-69747-z (PMC11324765; doi:10.1038/s41598-024-69747-z)
Supplement: Supplementary file 1 — Supplementary Information. [file 41598_2024_69747_MOESM1_ESM.pdf]

Supplementary Information

Constructing neural networks with pre-specified dynamics. *Camilo J. Mininni, B. Silvano Zanutto.*

Below we provide pseudocode for the gFTP algorithm and its key subfunctions, together with the following notes:

- The expression $x(1)$ represents the first element of a vector or list, while $x(end)$ is the last element.
- The expression $[x_1 ... x]$ stands for concatenation of $x_1 ... x_n$.
- The function DFS performs Depth First Search. When a cycle is encountered, the program stops and returns the arcs (v_s, v_t) that compose the cycle, and also a function *delta* that maps each arc with the $\delta_{i,j}$ that labels the arc.
- The graph D' is like graph D but with its arcs flipped when there were antiparallel superimposed arcs. It is equal to D if no antiparallel superpositions were found.
- The set V_{dif} is the set of all pairs of nodes that are differentiated by the neuron (their assigned z values are different).
- The difference between two sets A and B is written $A - B$.
- In Algorithm 1: gFTP, *max_iter* is the maximum number of iterations executed by the accelerated perceptron algorithm while searching for a solution. It was set to $1000 N_{tran}$.
- In Algorithm 3: Find_Superposition, when two sets in P are merged, the sets are removed from P, and a new set is added, that is the union of the two merged sets.
- In Algorithm 4: Make_Z_Target, the function z_{valid} takes a node as argument and returns a vector, specific for that node, which can be (1,0) or (0,1), chosen at random from a Bernoulli distribution. These vectors are defined once at the beginning of the algorithm, and are kept constant thereafter. The variable *firing_rate* is the parameter of the Bernoulli distribution. It was set to 0.5 in all experiments.

Algorithms were implemented in MATLAB R2021a (The MathWorks, Inc., Natick, MA, USA), running on a notebook computer with Intel^{TM} i7-8550U CPU and 32 GB RAM.

Algorithm 1 gFTP

Input: graph G

Output: graph G_{cons}, $\mathbf{Y}$, $\mathbf{Z}_s$, $\mathbf{Z}_t$, $\mathbf{W}$

$G_{cons} \leftarrow$**Make_Consistent**(G)

$e \leftarrow 1$

$\mathbf{Z}_t \leftarrow []$

Build $\mathbf{Y}$ by concatenating the identity matrix according to G_{cons}

while $e > 0$ **do**:

  pair_list$\leftarrow \{(v_i, v_j)\}_{j>i}$

  while *pair_list* $\neq \emptyset$ **do**:

    choose (v_i, v_j) from *pair_list*

    $\mathbf{z}, V_{dif} \leftarrow$ **Make_Z_Target**(G_{cons}, v_i, v_j)

    $\mathbf{Z}_t \leftarrow [\mathbf{Z}_t, \mathbf{z}]$

    remove V_{dif} from *pair_list*

  build $\mathbf{Z}_s$ from $\mathbf{Z}_t$ and G_{cons}

  $\mathbf{W}, e \leftarrow$ **Make_W**($\mathbf{Y}$, $\mathbf{Z}_s$, $\mathbf{Z}_t$, *max_iter*)

Algorithm 2 Make_Consistent

Input: G

Output: G

$D \leftarrow$ **Make_D**(G)

D', P, $V_{trav} \leftarrow$ **Find_Superposition**(D)

choose $C \in P$

choose $(v_s, v_t) \in C$

$D_{rest} \leftarrow D'$ restricted to $V_{tran}(C)$

while $D' \neq \emptyset$ **do:**

  cycle, delta $\leftarrow$ **DFS**(D_{rest}) starting in v_s

  If *cycle* $\neq \emptyset$:

    $G \leftarrow$ **Expand_Node**(G, *cycle*, *delta*)

    $D \leftarrow$ **Make_D**(G)

    D', P, $V_{trav} \leftarrow$ **Find_Superposition**(D)

    choose $C \in P$

    $D_{rest} \leftarrow D'$ restricted to $V_{trav}(C)$

  else:

    $D' \leftarrow D' - D_{rest}$

    $P \leftarrow P - C$

    choose $C \in P$

    choose $(v_s, v_t) \in C$

    $D_{rest} \leftarrow D'$ restricted to $V_{tran}(C)$

Algorithm 3 Find_Superposition

Input: D

Output: D_{output}, P, V_{trav}

$C_{used} \leftarrow \{\}$
$D_{output} \leftarrow D$
while $D \neq \emptyset$ **do**:
compute P, V_{trav} from D
choose $\mathscr{C} \in P$
$C_{comp} \leftarrow C - \mathscr{C}$
choose p, a complete path in $V_{trav}(\mathscr{C})$
$D_{und} \leftarrow$undirected version of D
for $v_i, v_j \in p$, $i \neq j$ **do**:
find path p_2 from v_i to v_j in D_{und}, through arcs in C_{comp}
choose arc a in p_2
find $C_a \in P$, such that the label of a is in C_a
merge C_a with C in P
update V_{trav} according to updated P
if a has opposite direction in p_2 than in D **and** label(a) $\notin C_{used}$:
flip the direction of all arcs in D_{output} that have labels in C_a
$C_{used} \leftarrow C_{used} \cup C_a$
remove p from D

Algorithm 4 Make_Z_Target

Input: G, v_1, v_2

Output: $\mathbf{z}$, V_{dif}

$(\mathbf{z}(t_1), \mathbf{z}(t_2)) \leftarrow (1,0)$ with $(f_t(t_1), f_t(t_2)) = (v_1, v_2)$
def: $z_{valid} : v \rightarrow [x, 1-x], x \sim$ Be(*firing_rate*)
$z^0_{valid} \leftarrow z_{valid}$
$V \leftarrow$nodes in G, sorted in order of decreasing in-degree
$V_{def} \leftarrow [v_1, v_2]$
$L_u \leftarrow V - V_{def}$
$L_a \leftarrow L_u(1)$
$\mathbf{z} \leftarrow$empty vector of dimension N_{tran}
$\mathbf{z}_{backup} \leftarrow [\,]$
while $L_a \neq \emptyset$ **and** $L_u \neq \emptyset$ **do**:
    $v \leftarrow L_a(end)$
    $z_1 \leftarrow z_{valid}(v)$
    if $z_1 \neq \emptyset$ **do**:
        $z \leftarrow z_1(1)$
        $\mathbf{z}^0 \leftarrow \mathbf{z}$
        $\mathbf{z}_{backup}(v) \leftarrow \mathbf{z}$
        $\mathbf{z}(t) \leftarrow z$ for transitions t in which v is a target node
        remove z from $z_{valid}(v)$
        consistency $\leftarrow$ **Check_Consistency**(D, $\mathbf{z}$)
        if *consistency* = True:
            propagate deltas over $\mathbf{z}$
            consistency $\leftarrow$ **Check_Consistency**(D, $\mathbf{z}$)
            if *consistency* = True:
                $V_{def} \leftarrow$ [v with z value assigned in $\mathbf{z}$]
                $L_u \leftarrow V - V_{def}$
                $L_a \leftarrow [L_a, L_u(1)]$
            else:
                $\mathbf{z} \leftarrow \mathbf{z}^0$
        else:
            $\mathbf{z} \leftarrow \mathbf{z}^0$
    else:
        $L_a \leftarrow L_a - v$
        $\mathbf{z} \leftarrow \mathbf{z}_{backup}(L_a(end))$
        $V_{def} \leftarrow$ [v with z value assigned in $\mathbf{z}$]
        $L_u \leftarrow V - V_{def} - L_a(end)$
        reset z_{valid} to z^0_{valid} for each $v \in L_u$
$V_{dif} \leftarrow \{(v_i, v_j)\}_{ij}$ s.t. $z(v_i) \neq z(v_j)$

Algorithm 5 Expand_Node

Input: G, *cycle, delta*

Output: G

1. choose $e_{cycle} = (v_1, v_2)$, an arc in *cycle*
2. $\delta_{s_1,s_2} \leftarrow delta(e_{cycle})$
3. $v_{replace} \leftarrow e_{cycle}(1)$
4. $v_{new} \leftarrow |V| + 1$
5. **for** all pairs of arcs $a_1 \leftarrow (v_s, v_{t_1})$, $a_2 \leftarrow (v_s, v_{t_2})$ in G **do**:
6. **if** $(v_{t_1}, v_{t_2}) = e_{cycle}$ and $(\text{label}(a_1), \text{label}(a_2)) = (s_1, s_2)$:
7. delete arc a_2 with label s_2 in G
8. add arc (v_s, v_{new}) to G
9. **for** each arc $a \leftarrow (v_{source}, v)$ in G with $v_{source} = v_{replace}$ **do**:
10. add to G an arc (v_{new}, v_{target}) with the label of a
